# Supplementary figures and images for: Role of Rabenosyn-5 and Rab5b in host cell cytosol uptake reveals conservation of endosomal transport in malaria parasites
Source: PLoS Biol. 2024 May 31;22(5):e3002639. doi: 10.1371/journal.pbio.3002639 (PMC11168701; doi:10.1371/journal.pbio.3002639)

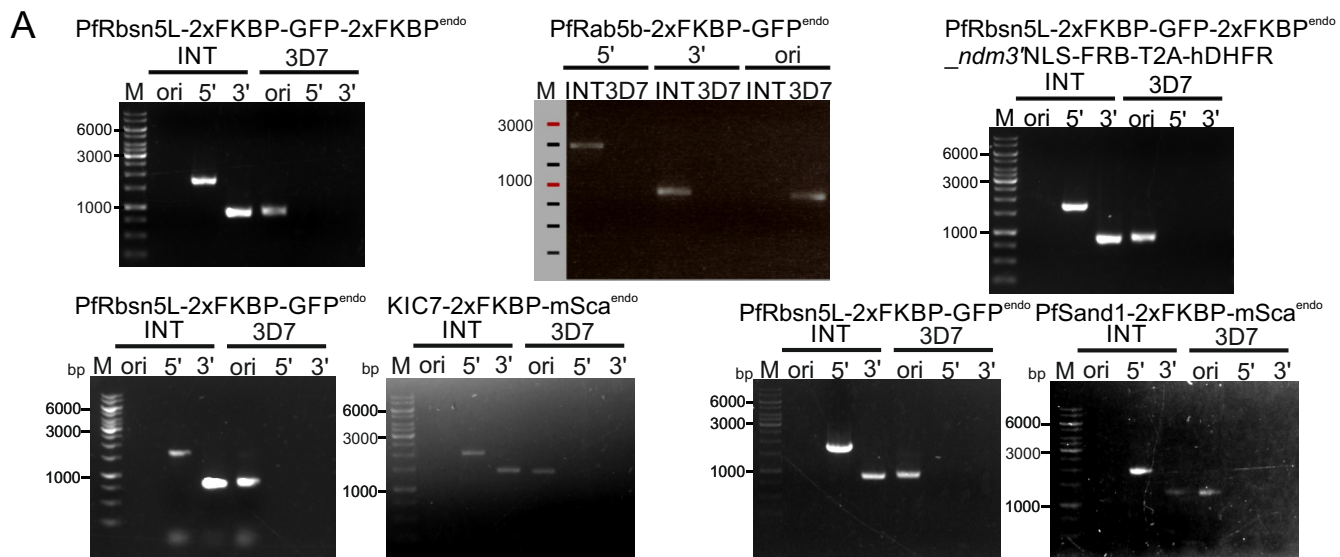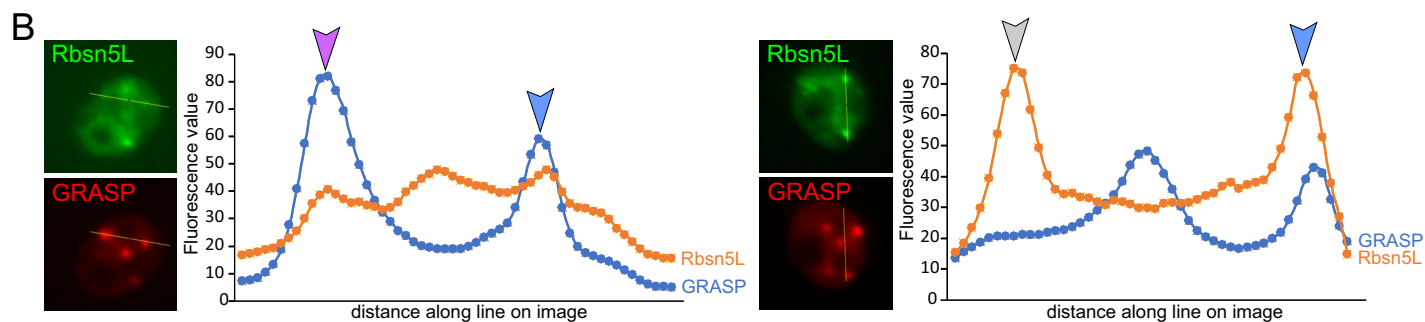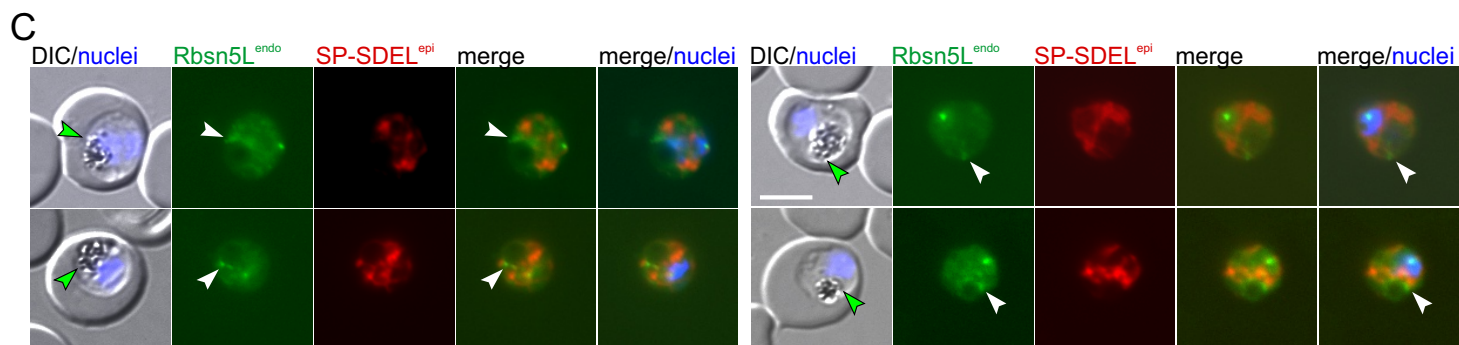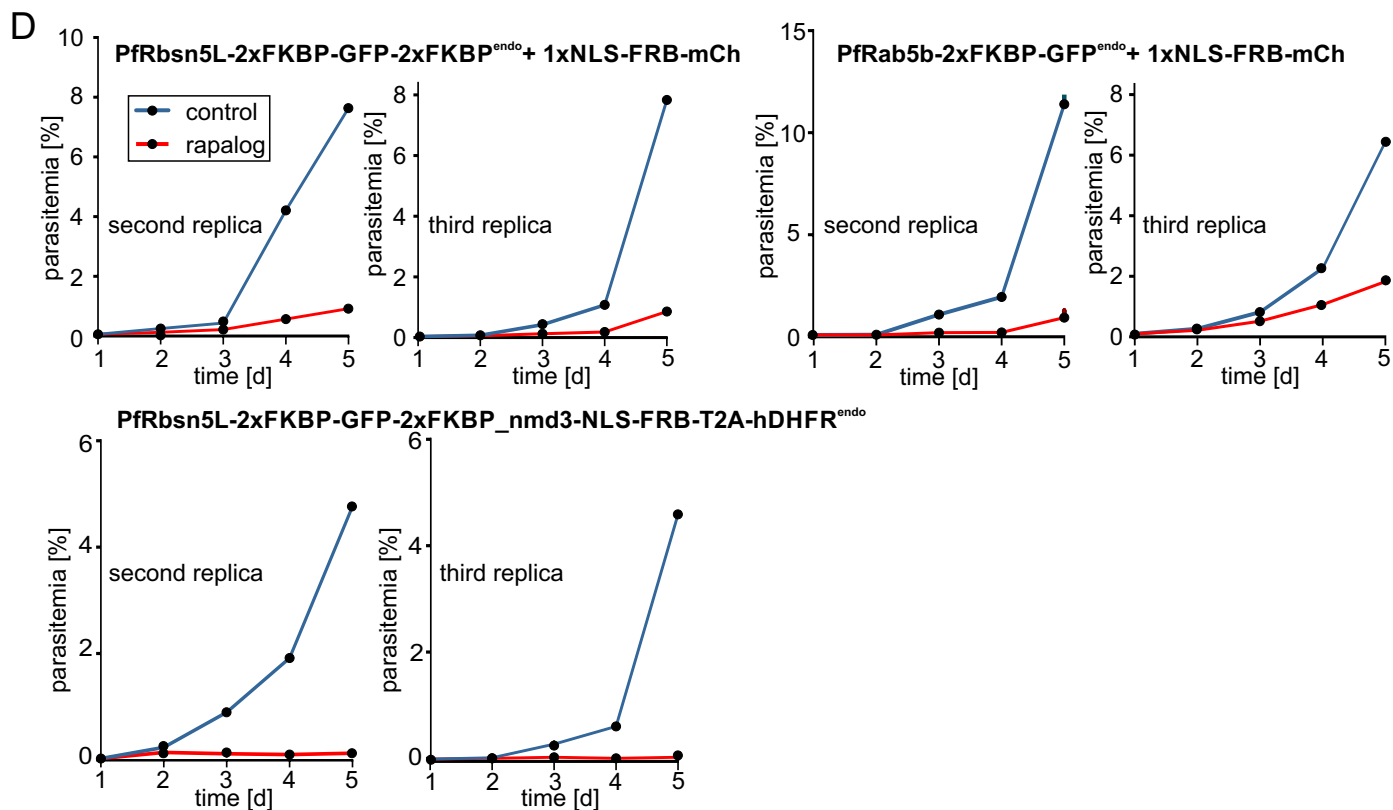

Supplement: S2 Fig — (A) Agarose gels showing PCR-products to assess correct integration of the SLI-plasmids into the genome of P. falciparum (3D7) parasites to obtain the indicated cell lines (see also S1 Raw Images). 3D7, parent cell line; INT, integration cell line; 5′, PCR product across the 5′ integration junction; 3′, PCR product across the 3′ integration junction; ori, original locus (absence showing lack of parasites with unmodified locus). M, marker with selected fragments indicated in bp. Primers used for integration confirmation and expected sizes of PCR products listed in S1 Table and sequences of the plasmids in S1 File. (B) Intensity profiles along the indicated lines in images from Fig 1C generated with ImageJ. Lines were drawn in both images using the synchronize images command and the plot profile values were used to draw graphs in Excel. Arrows show green foci and are color coded as in Fig 1C. (C) Live-cell microscopy images of PfRbsn5L-2xFKBP-GFP-2xFKBPendo parasites, co-expressing the ER-marker STEVOR-SP-mScarlet-SDLepi (SP-SDELepi). White arrows show PfRbsn5Lendo foci close to the food vacuole that were added at the same position in the DIC/Hoechst image as green arrows to illustrate the position relative to the food vacuole. (D) Replicates of the flow cytometry growth curves shown in the main figures (cell lines indicated). The data underlying the growth curve replicates can be found in S1 Data under the main figure designation of the respective growth curve. (PDF) [file pbio.3002639.s002.pdf]

A

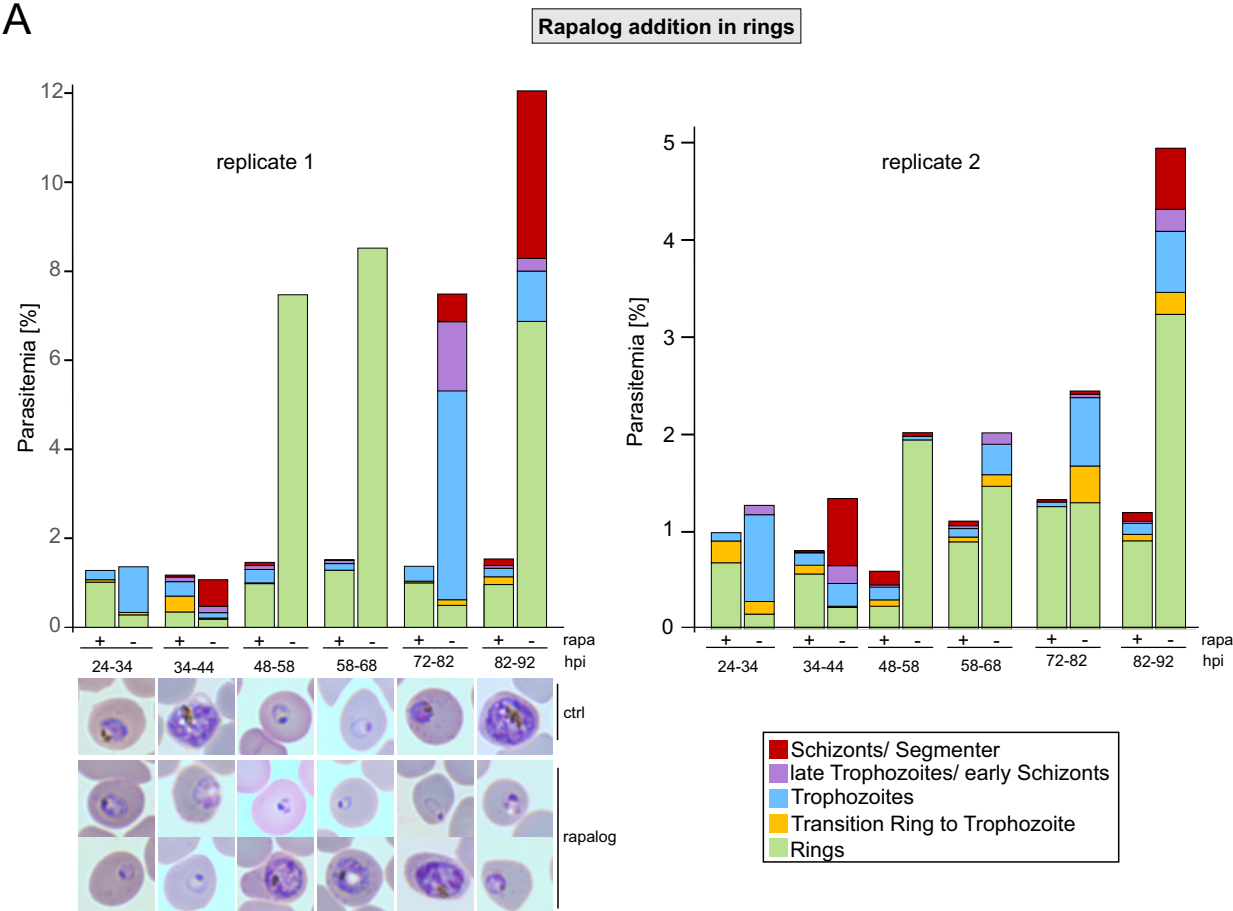

B

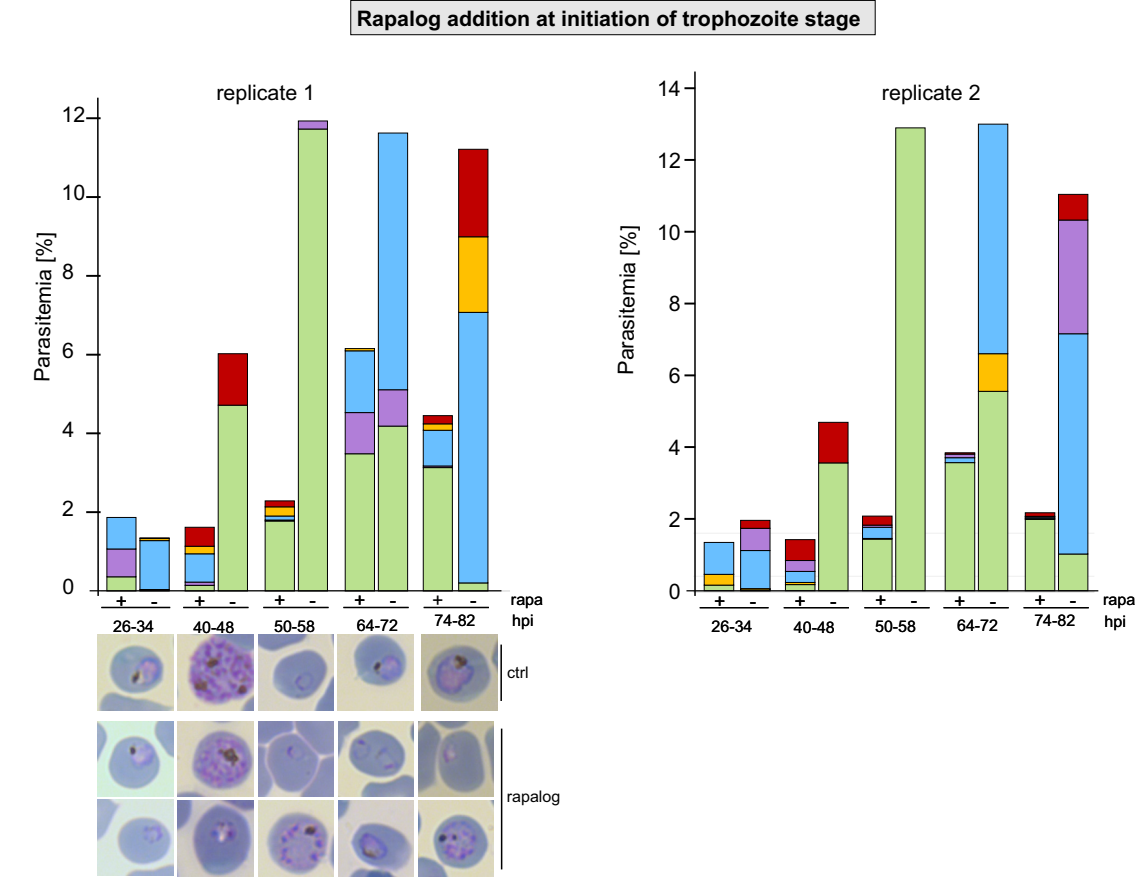

Supplement: S3 Fig — (A, B) Synchronous parasites were inactivated in rings (A) or at the start of the trophozoite stage (B) (see Materials and methods) and the development monitored based on Giemsa smears. The rings monitored correspond to parasites where PfRbsn5L was inactivated 0–8 h post invasion (hpi), the trophozoites at 16–24 hpi (n = 2 independent experiments; Giemsa smears shown only for replicate 1). Note that cells scored as rings after prolonged growth arrest in + rapa in the ring induction showed various morphological alterations not specifically scored. The data underlying this figure can be found in S1 Data. (PDF) [file pbio.3002639.s003.pdf]

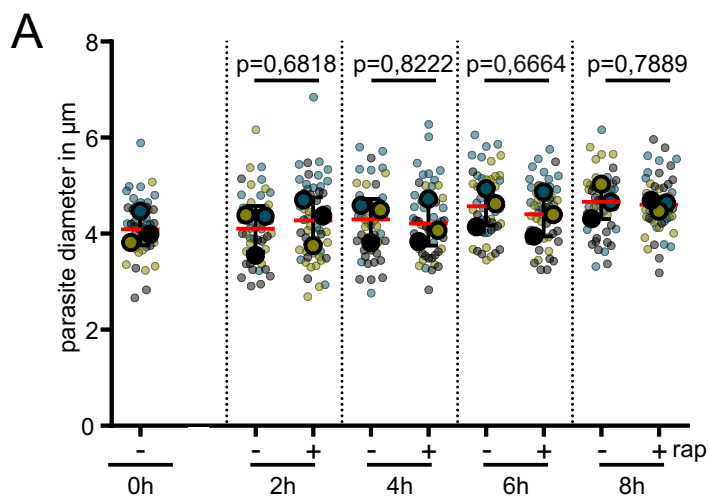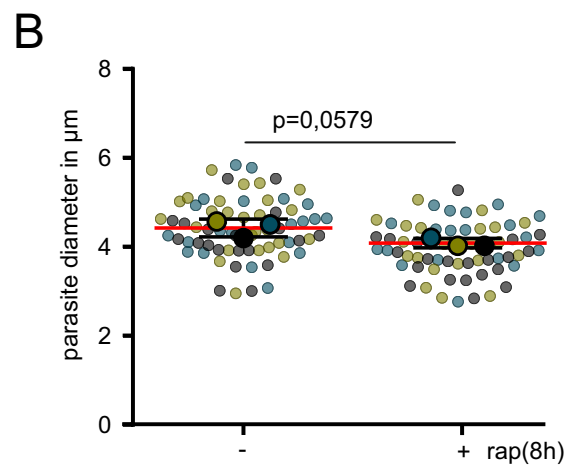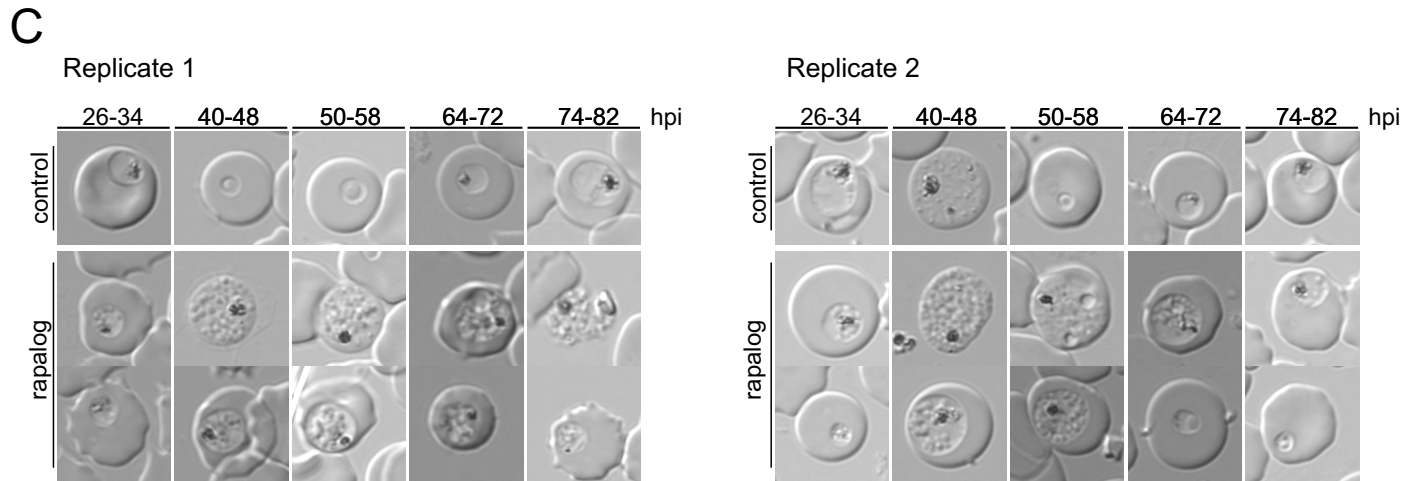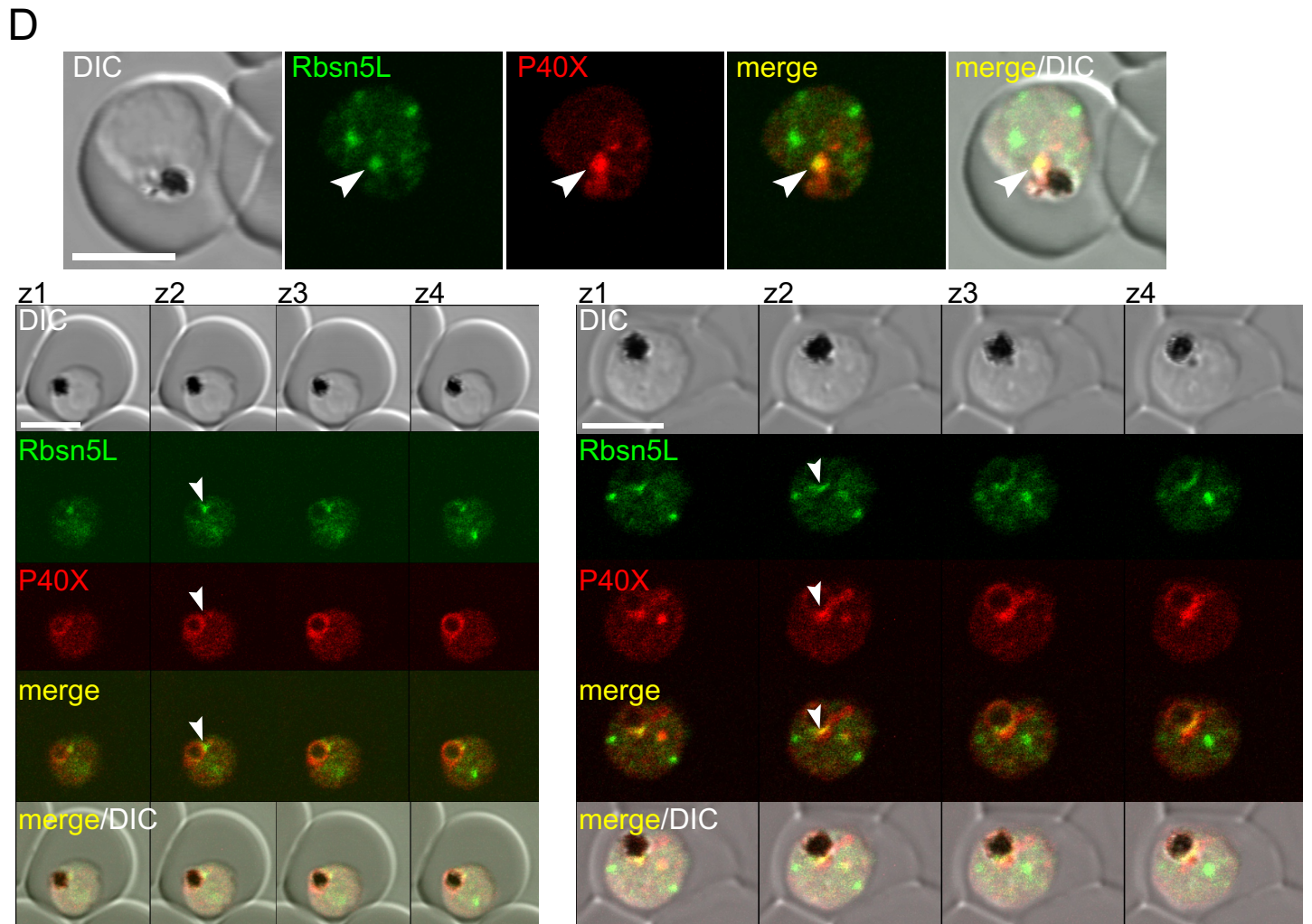

Supplement: S4 Fig — (A, B) Superplots showing diameter in μm of parasites analyzed in the vesicle accumulation assay in Fig 1G (A) and of parasites analyzed in the bloated FV assay in Fig 2B (B). Parasites from n = 3 independent experiments are distinguished by blue, yellow, and black dots; two-tailed unpaired t test of the means, p-values indicated; mean (red bar); error bars (black) show SD. (C) DIC example images showing the phenotype of time points of the parasites from the stage growth assay in S3 Fig that resulted in the growth phenotype (time points indicated). (D) Confocal microscopy images of PfRbsn5Lendo parasites (PfRbsn5L: green channel) co-expressing the mScarlet tagged PI3P marker P40X (red channel) used for the experiments shown in Fig 2E. Top, single z-slice, bottom panels show 4 consecutive confocal z-slices (z1-4). White arrows: PI3P positive regions overlapping with PfRbsn5L accumulations adjacent or at the food vacuole. DIC, differential interference contrast; merge, overlay of red and green channels. Size bars, 5 μm. The data underlying this figure can be found in S1 Data under the main figure indicated for each figure part. (PDF) [file pbio.3002639.s004.pdf]

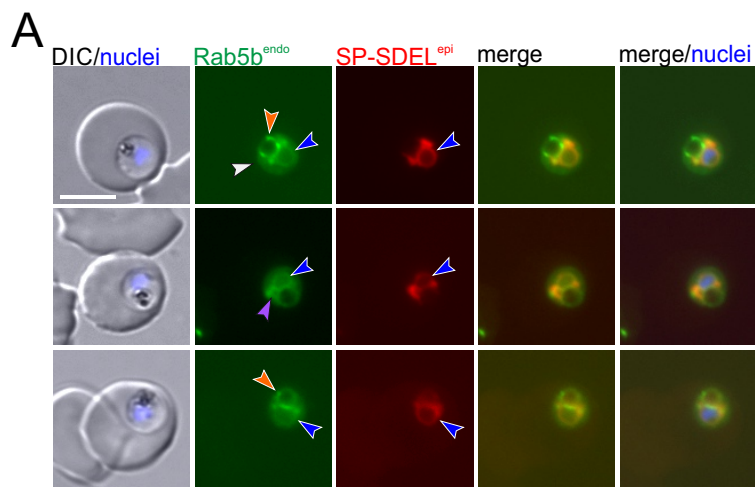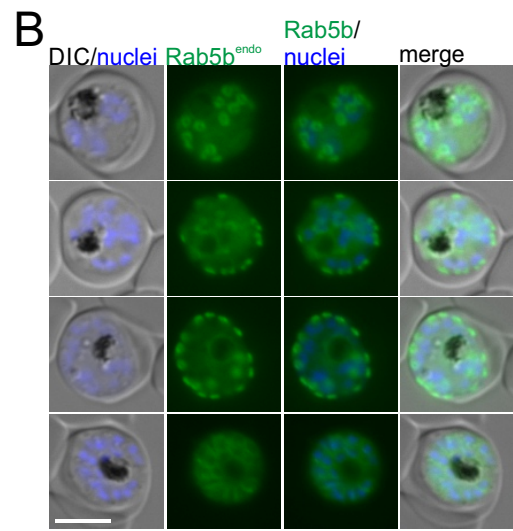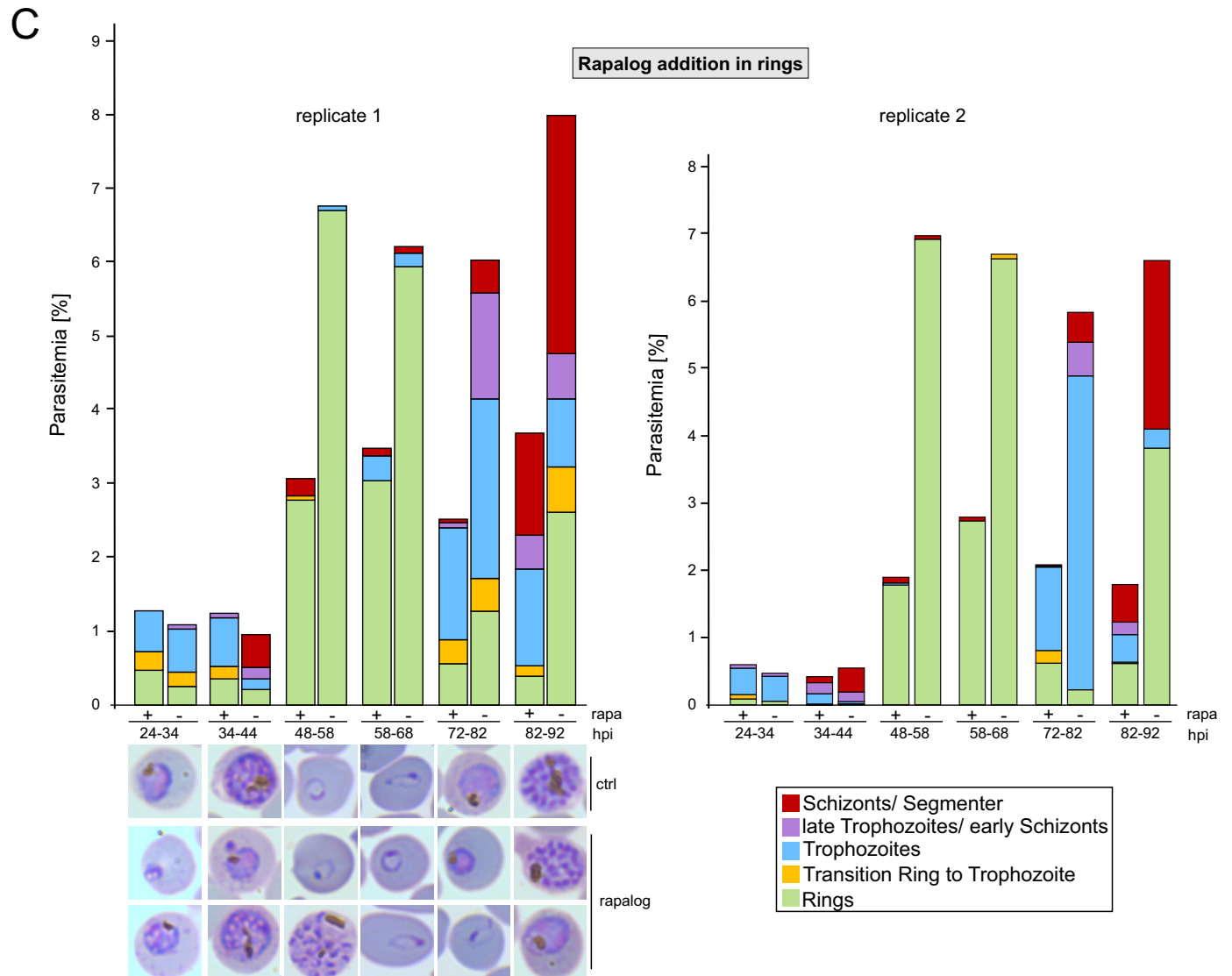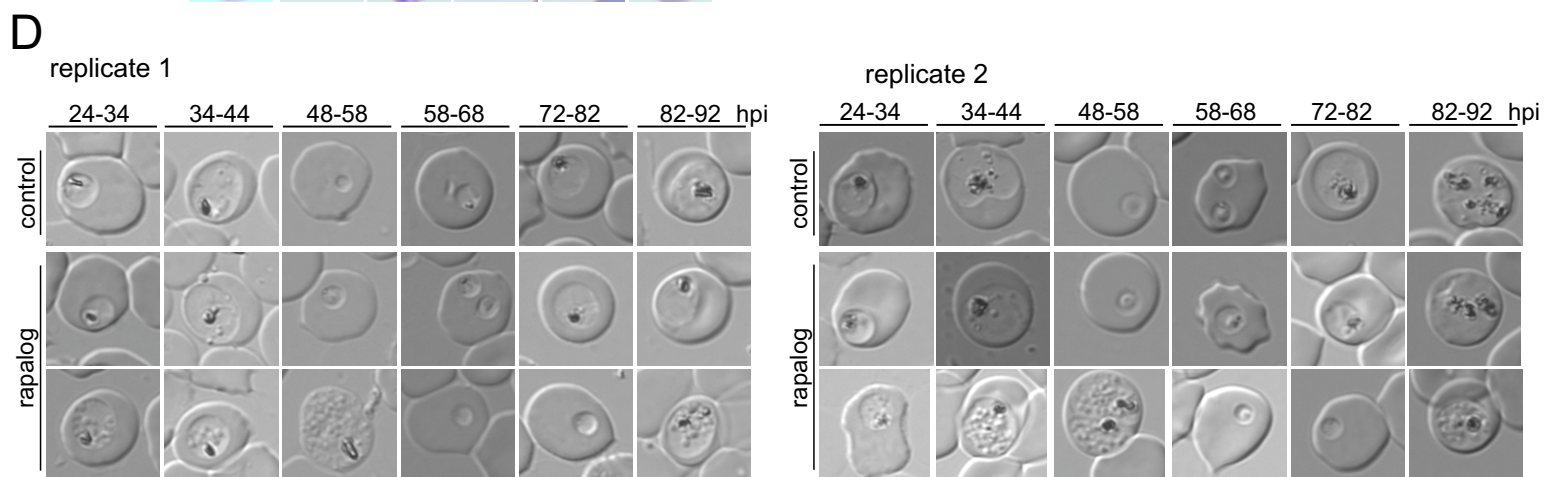

Supplement: S5 Fig — (A, B) Live-cell microscopy images of PfRab5bendo parasites co-expressing the ER marker STEVOR-SP-mScarlet-SDLepi (SP-SDELepi) (A), and of PfRab5bendo schizonts (B) showing a location suggestive of the IMC. Merge, overlay of green and red channels. Arrows in A show: blue, ER membrane; white, parasite plasma membrane; orange, Rab5b foci at the FV not overlapping with the ER marker; purple, possible FV loop-like structure. Size bar 5 μm. Nuclei were stained with DAPI. (C) Growth assay after conditional inactivation of Rab5b in synchronous rings monitoring progression through the cycle based on Giemsa smears (the parasites monitored correspond to parasites where Rab5b was inactivated 0–8 h post invasion (hpi)) (n = 2 independent experiments). Example Giemsa smear images shown for replicate 1. (D) DIC example images showing the phenotype in the parasites from the stage growth assay in C of the time points (indicated) leading to the growth phenotype. The data underlying this figure can be found in S1 Data. (PDF) [file pbio.3002639.s005.pdf]

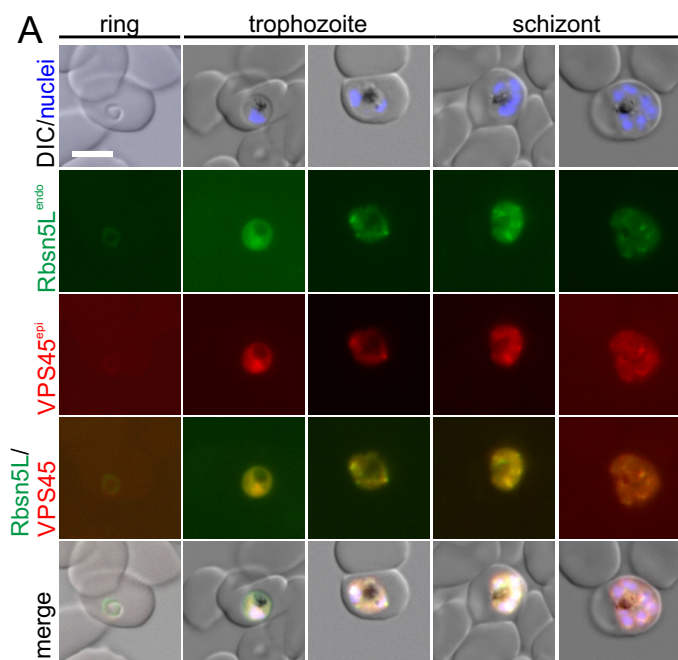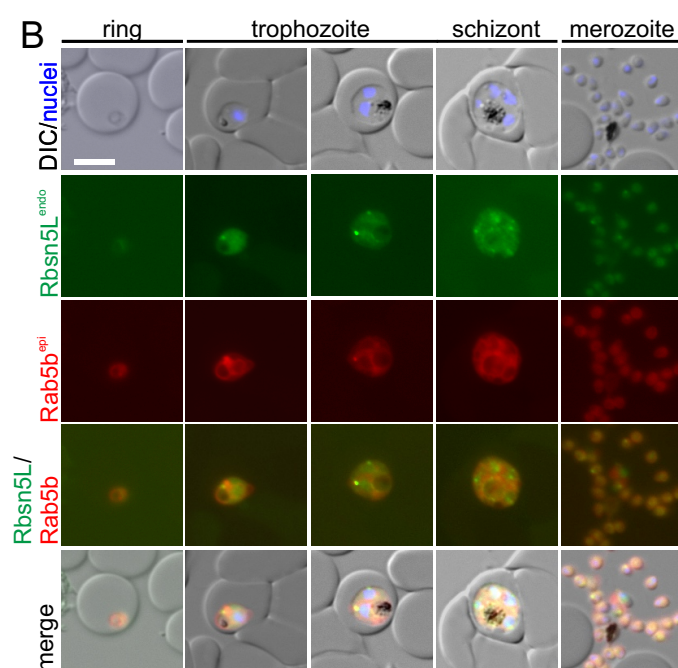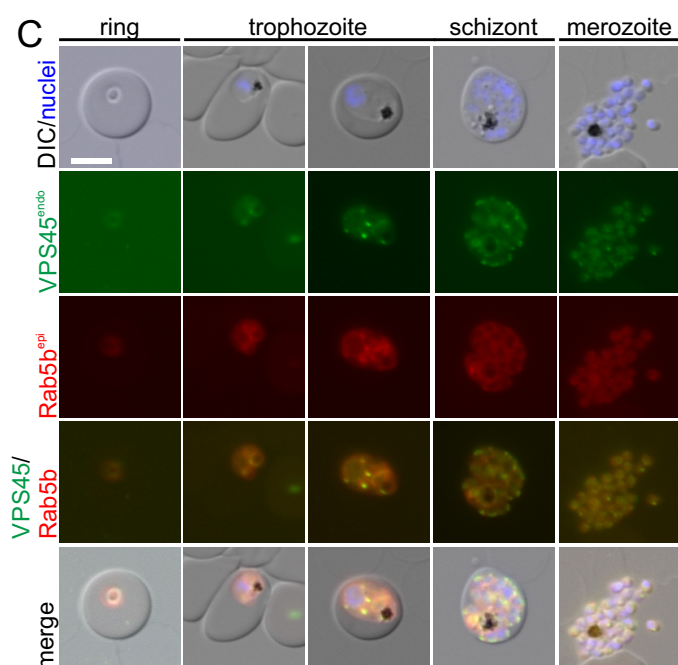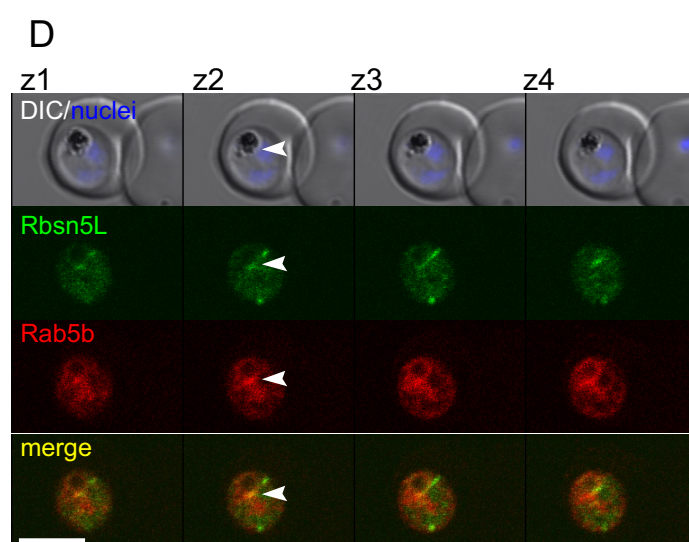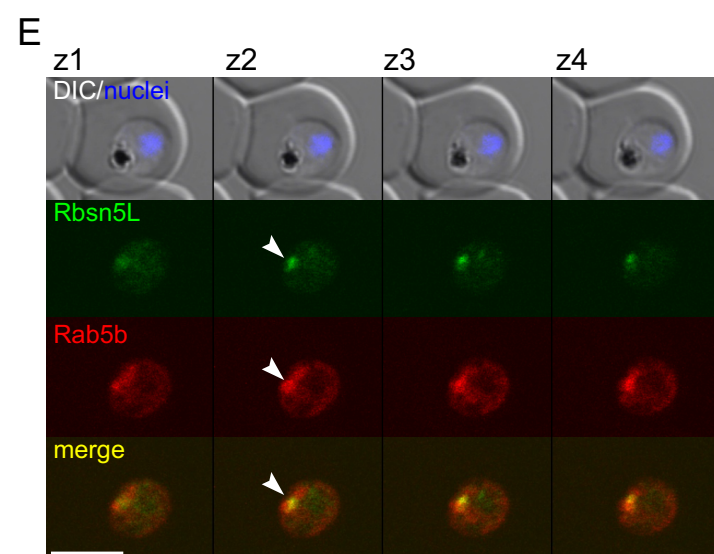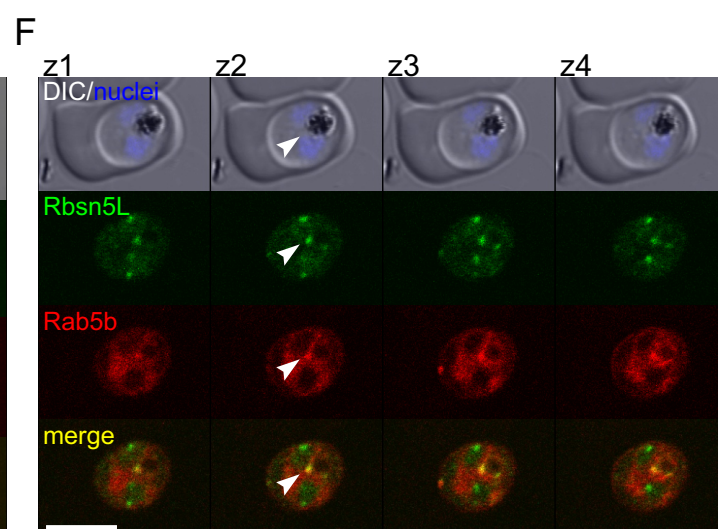

Supplement: S6 Fig — (A–C) Live-cell microscopy images of the indicated stages of PfRbsn5L-2xFKBP-GFP-2xFKBPendo parasites, co-expressing PfVPS45-mChepi (A), PfRbsn5L-2xFKBP-GFP-2xFKBPendo parasites, co-expressing PfRab5b-mChepi (B), and PfVPS45-2xFKBP-GFPendo parasites, co-expressing PfRab5b-mChepi (C). DIC, differential interference contrast; endo, endogenous; epi, episomal. Scale bar, 5 μm. Nuclei were stained with DAPI. (D–F) Confocal microscopy images of PfRbsn5Lendo + Rab5bepi parasites (PfRbsn5L: green channel; Rab5b: red channel) showing 4 consecutive confocal z-slices (z1-4) per cell. White arrows: Regions where PfRbsn5L overlaps with Rab5b. Nuclei were stained with Hoechst; DIC, differential interference contrast; merge, overlay of red and green channels. Size bars, 5 μm. (PDF) [file pbio.3002639.s006.pdf]

CoIP with PfRbsn5-2xFKBP-GFP-2xFKBP<sup>endo</sup> + PfVPS45-mCh<sup>epi</sup> parasites

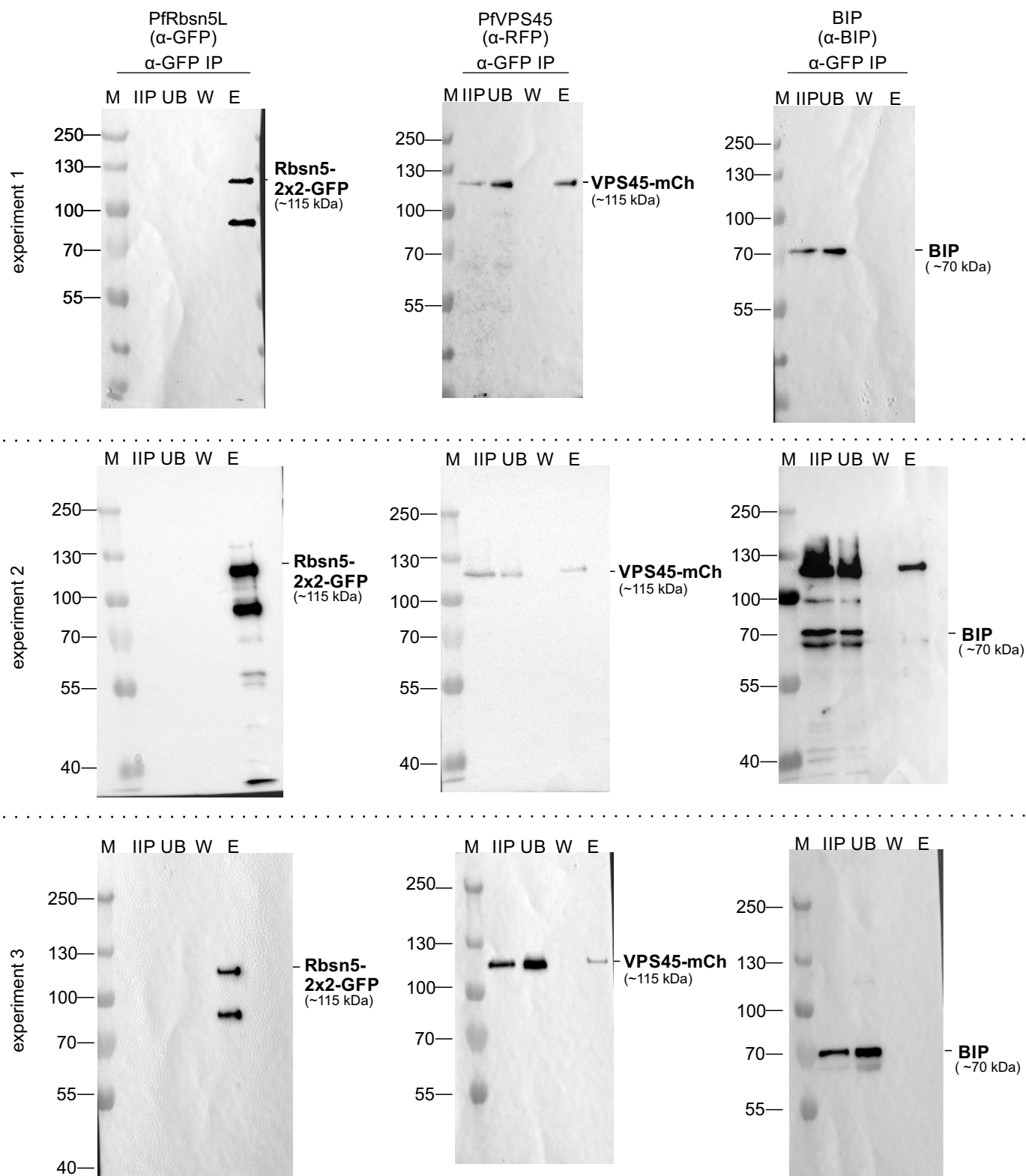

Supplement: S7 Fig — Replicas and complete blots of 3 independent experiments of Immunoprecipitation (IP) of extracts from PfRbsn5Lendo parasites co-expressing PfVPS45epi (one cropped version is shown in Fig 5D). Note that only the indicated band on the anti-BIP blot in experiment 2 corresponds to BIP, the other bands are from a previous probing of the same membrane. IIP, IP-input extract; UB, unbound (total extract after IP); W, last wash; E, eluate; endo, endogenous; epi, episomal. See S1 Raw Images for uncropped blots merged with marker as obtained directly from the ChemiDoc XRS imaging system. (PDF) [file pbio.3002639.s007.pdf]

CoIP with PfRbsn5L-2x2FKBP-GFP-2x2FKBP<sup>endo</sup> + PfRab5b-mCh<sup>epi</sup> parasites

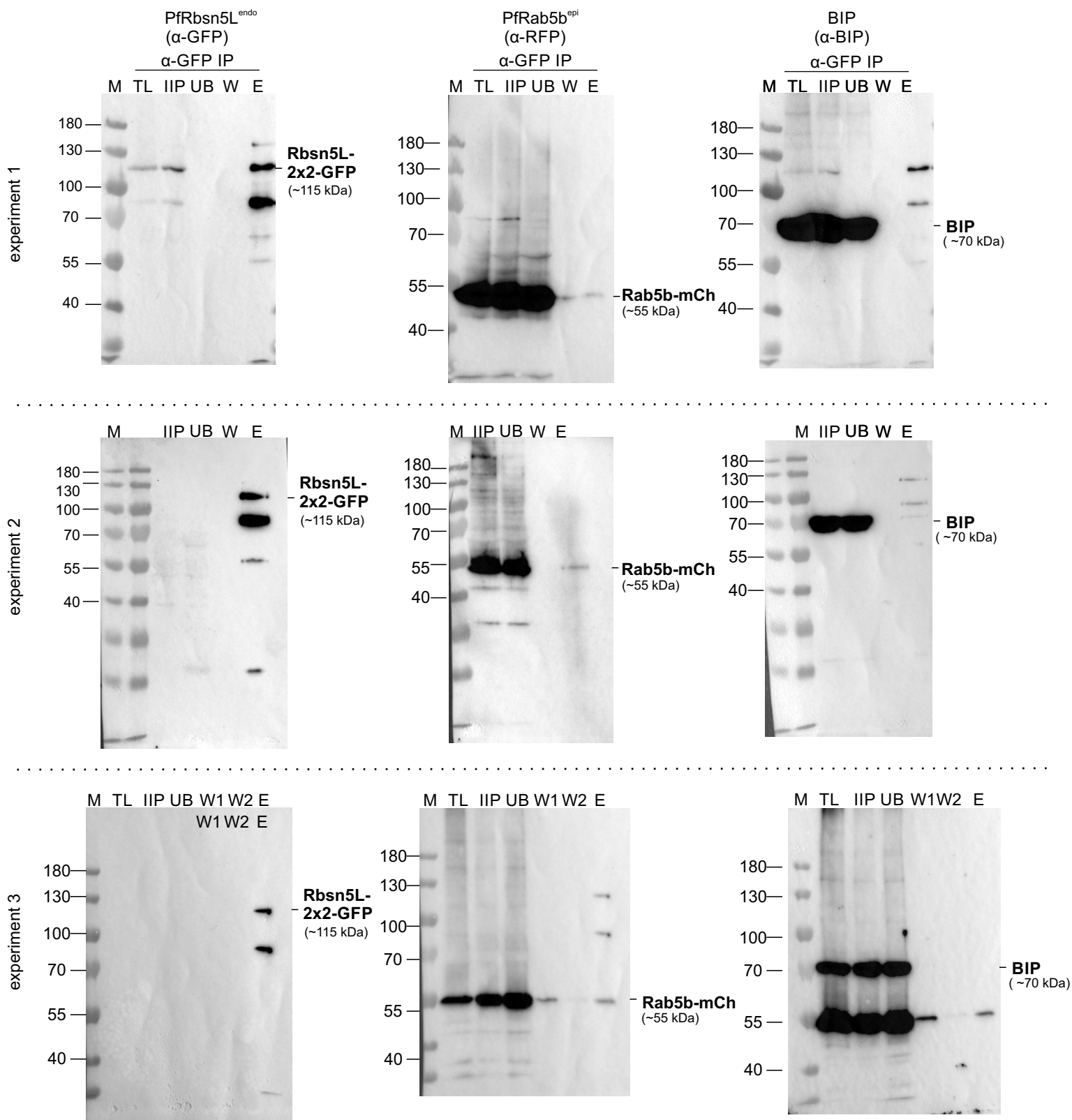

Supplement: S8 Fig — Replicas and complete blots of 3 independent experiments of Immunoprecipitation (IP) of PfRbsn5Lendo parasites co-expressing PfRab5bepi (one cropped version is shown in Fig 5E). Note that only the indicated band on the anti-mCherry and anti-BIP blots in experiment 3 correspond to the respective proteins, the other bands are from a previous probing of the same membrane. IIP, IP-input extract; UB, unbound (total extract after IP); W, last wash; W1, first wash; W2, second wash; E, eluate; endo, endogenous; epi, episomal. See S1 Raw Images for uncropped blots merged with marker as obtained directly from the ChemiDoc XRS imaging system. (PDF) [file pbio.3002639.s008.pdf]

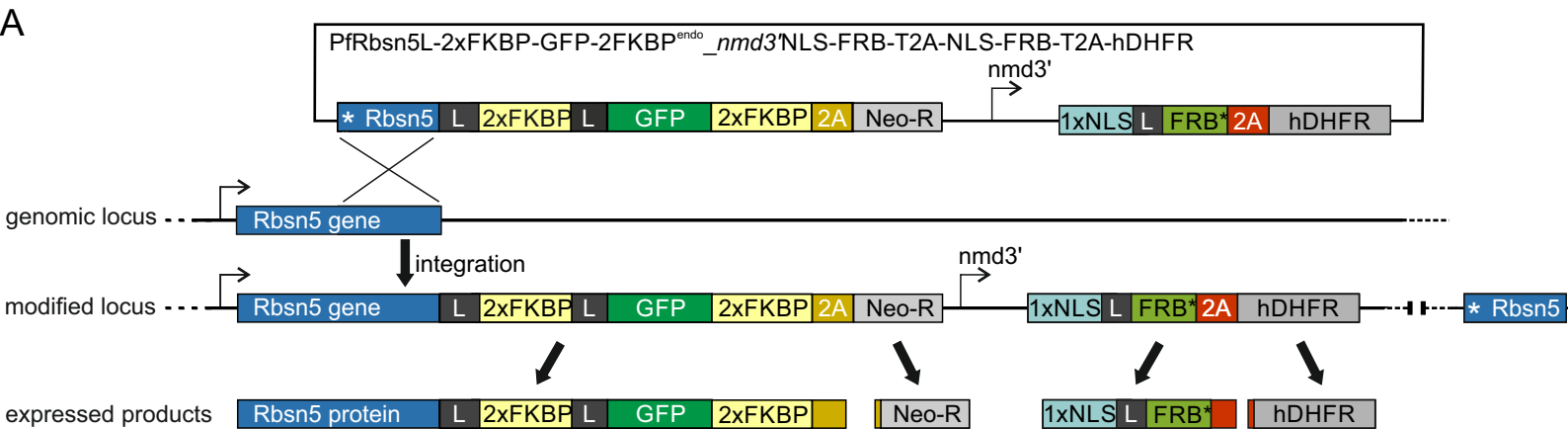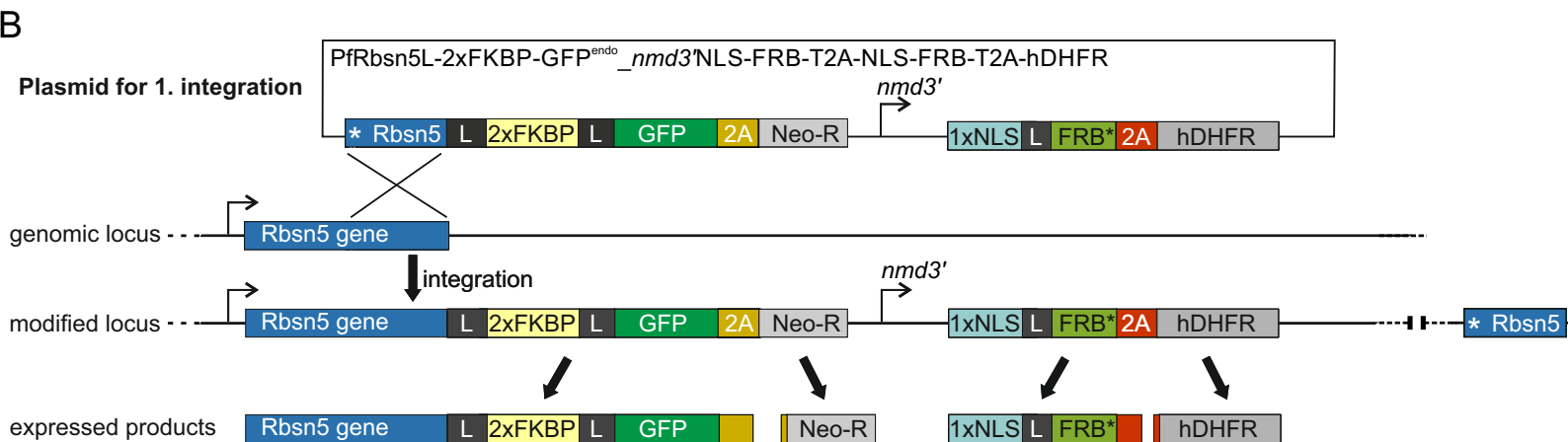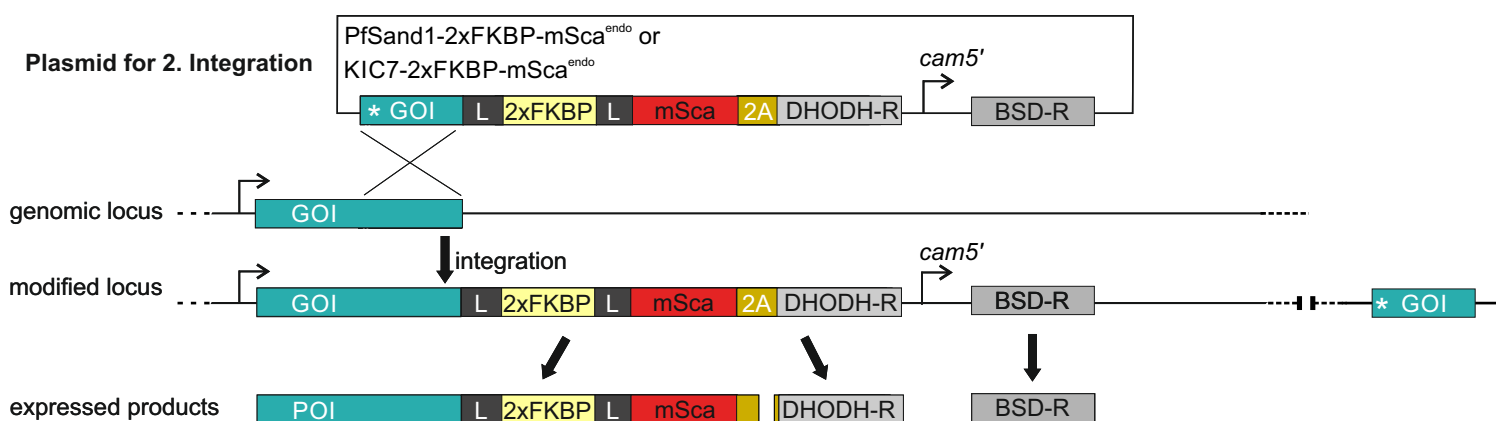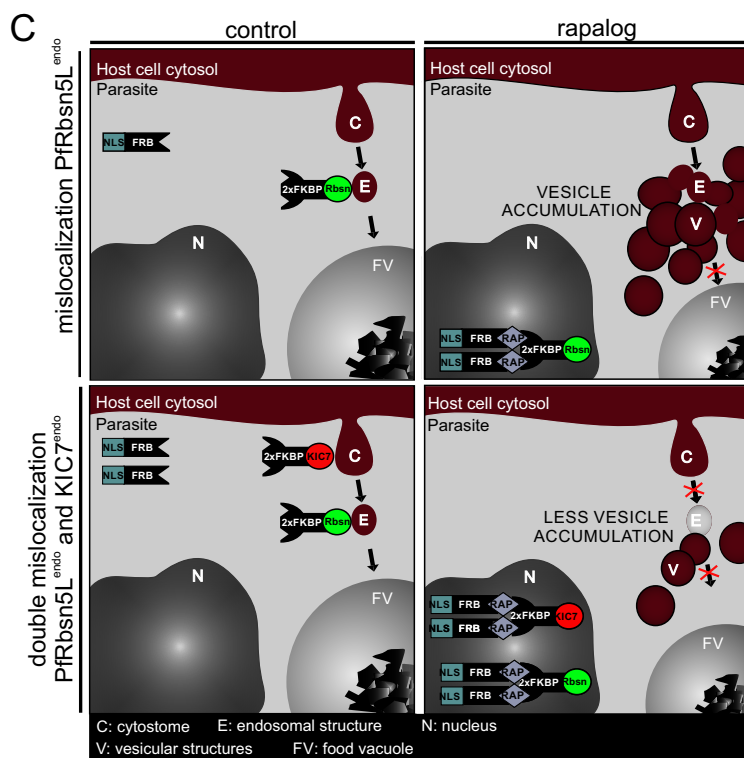

Supplement: S9 Fig — (A) Schematic of selection linked integration strategy combined with a mislocalization cassette on the same plasmid. (B) Scheme of a selection linked integration strategy enabling endogenously tagging and simultaneous mislocalization of 2 POIs in the same parasites. (C) Schematic illustration of simultaneous double mislocalization of KIC7 (which inhibits endocytosis at the PPM in an early step) and PfRbsn5L (which inhibits transport of HCC-filled vesicles to the food vacuole), showing the expected reduction in vesicle accumulation compared to PfRbsn5L inactivation alone if the 2 processes are serially linked. (PDF) [file pbio.3002639.s009.pdf]
