# Supplementary material for: Role of Rabenosyn-5 and Rab5b in host cell cytosol uptake reveals conservation of endosomal transport in malaria parasites
Source: PLoS Biol. 2024 May 31;22(5):e3002639. doi: 10.1371/journal.pbio.3002639 (PMC11168701; doi:10.1371/journal.pbio.3002639)

A **FYVE domains**

Logo of motifs

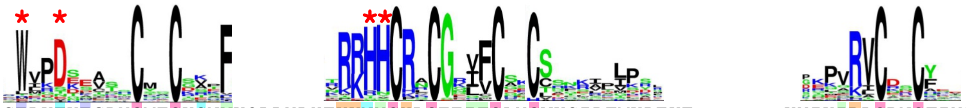

```
Pf_Rbsn5L         44 SELKTKEGDYCMTCKSNVKQLLYLHTKKNYCHLCEEIFCAYCVKSIDFMKDEKE-------KYIKIRLCRNCFIYIN  119
PF3D7_1460100     32 LWVPDEEVTNCYSCNVVF----NVRVRKHHCRACGNVFCSNCSDNKIKISEYSYS--------EKVRVCDRCFMERS   96
human_Protrudin  122 KRYPTNNFGNCTGCSATF----SVLKKRRSCSNCGNSFCSRCCSFKVPKSSMGAT--APEAQRETVFVCASCNQTLS  192
yeast_PEP7       206 PWRDDRSVLFCNICSEPF----GLLLRKHHCRLCGMVVCDDANRNCSNEISIGYL-20aa-IPISIRLCSHCIDMLF  297
human_EEA1      1298 KWAEDNEVQNCMACGKGF----SVTVRRHHCRQCGNIFCAECSAKNALTPSSKK----------PVRVCDACFNDLQ 1411
human_Rbsn5      147 PWVNDQDVPFCPDCGNKF----SIRNRRHHCRLCGSIMCKKCMELISLPLANKLT-43aa-DDDRIRCCTHCKDTLL  260
human_FYV1       148 YWMPDSQCKECYDCSEKF----TTFRRRHHCRLCGQIFCSRCCNQEIPGKFMGYT—-------GDLRACTYCRKIAL  218
```

Positively charged
Negatively charged
Polar Uncharged
Hydrophobic
Cysteine
* residues critical for PI3P binding that do not follow consensus in Pf_Rbsn5L

B **Rab-binding domains (RabBD)**

```
                                 +       +              ++      +
PF_Rbsn5          130 ----SVDRKAIDLQNSFNDISNCYTNLCSNVPQLNGLVLLCENNKEFLDSFKSEIKQLEE 195
yeast_PEP7        464 --------VLKE---QSYLI-ESTIQDYKKQRRLEEIVTLNKNLEELHSRIH-------- 503
Drosophila_Rbsn5  441 ASKSSLDDPLIE---QINII-KGYIKQARQDMNFEVVETLELNLRELQREVYERQRQS-- 494
human_Rbsn5-copy1 458 --------PLLQ---QIHNI-TSFIRQAKAAGRMDEVRTLQENLRQLQDEYDQQ------ 499
human_Rbsn5-copy2 739 ---------LLQ---QIDNI-KAYIFDAKQCGRLDEVEVLTENLRELKHTLAKQ------ 779
                                 :   .   *            .:: :  *  *  .::
```

Positively charged
Negatively charged
Polar Uncharged
Hydrophobic
Cysteine
+ involved in conserved hydrogen bonds in human Rbsn5

C

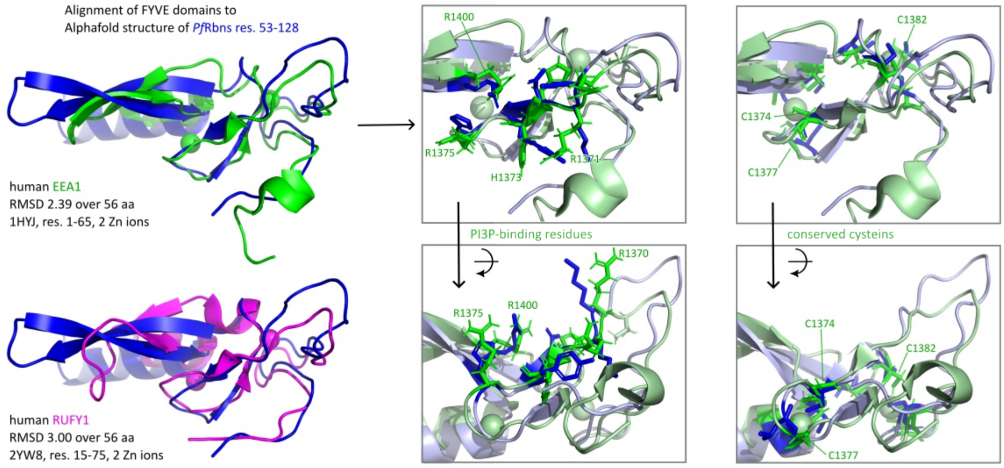

D

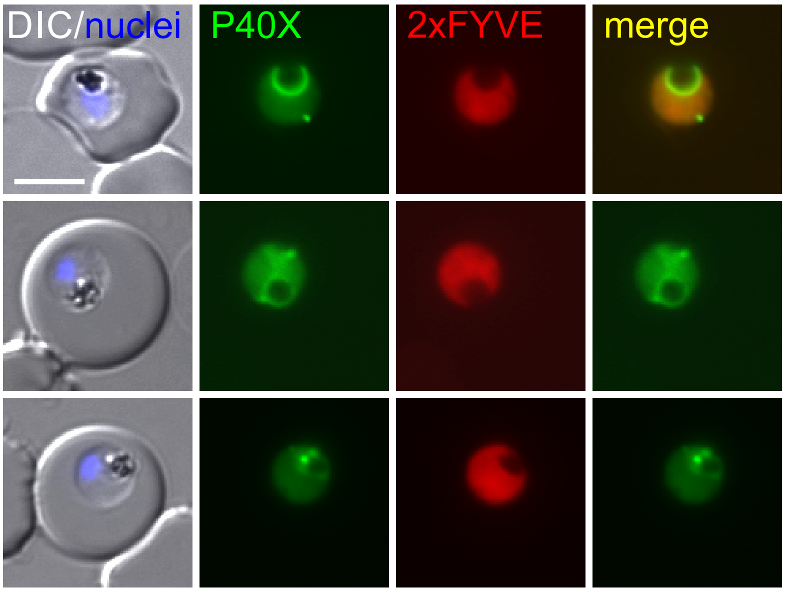

Supplement: S1 Fig — (A, B) Multiple sequence alignment of (A) FYVE and (B) Rab-binding domains of the indicated proteins and regions. Residues indicated as conserved were colored according to their physical properties. Sequence logo of the conserved residues in A was generated from the sequences of all FYVE domains found in H. sapiens, S. cerevisiae, T. brucei, A. thaliana, G. intestinalis, P. falciparum, and T. gondii. Red asterisks show amino acids important for PI3P binding or specificity that are not conserved in the FYVE domains of PfRbsn5L and human Protrudin (which contains an FYVE domain not binding PI3P but other phosphoinositides) while they are conserved in PF3D7_1460100 (FCP), the likely PfEEA1 [36]. In B, similarity according to clustal omega (dots and asterisks) are indicated below the alignment; residues involved in hydrogen bonds that are conserved between both Rab-binding domains of human Rbsn5, according to PDB 1z0k and 1z0j, were marked with + above. (C) Structural alignment of AlphaFold2 predicted structure of PfRbsn5L FYVE domain compared to the experimental structures of human EEA1 and human RUFY1. (D) Expression of a tandem of the PfRbsn5L FYVE (2xFYVE) domain fused to mCherry in parasites expressing P40X-GFP (P40X) to mark PI3P positive regions. Nuclei were stained with DAPI; DIC, differential interference contrast; merge: overlay of green and red channels; size bar: 5 μm. (PDF) [file pbio.3002639.s001.pdf]
