# Supplementary material for: Role of Rabenosyn-5 and Rab5b in host cell cytosol uptake reveals conservation of endosomal transport in malaria parasites
Source: PLoS Biol. 2024 May 31;22(5):e3002639. doi: 10.1371/journal.pbio.3002639 (PMC11168701; doi:10.1371/journal.pbio.3002639)

Agarose gels shown in S2A Fig

PfRbsn5L-2xFKBP-GFP-2xFKBP^{endo}

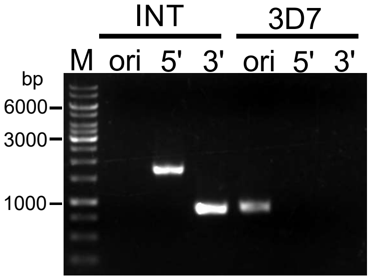

PfRab5b-2xFKBP-GFP^{endo}

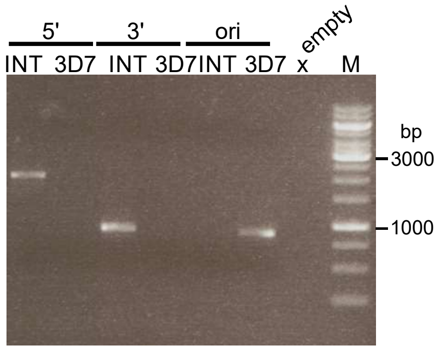

PfRbsn5L-2xFKBP-GFP-2xFKBP^{endo}
_*ndm3'*NLS-FRB-T2A-hDHFR

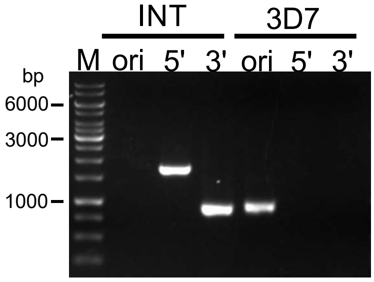

PfRbsn5L-2xFKBP-GFP^{endo}

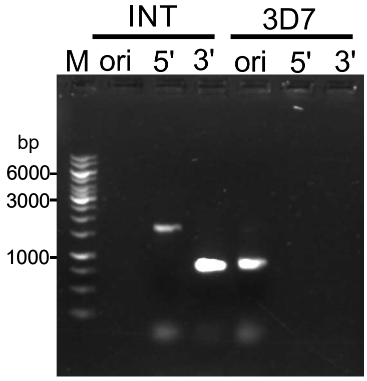

KIC7-2xFKBP-mSca^{endo}

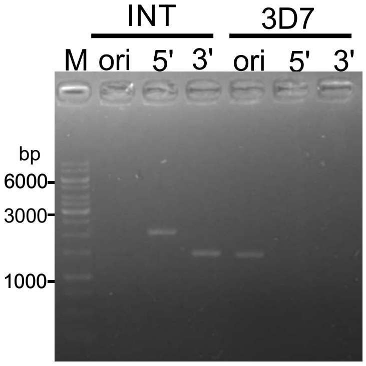

PfRbsn5L-2xFKBP-GFP^{endo}

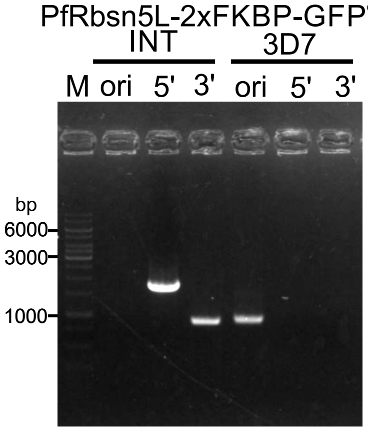

PfSand1-2xFKBP-mSca^{endo}

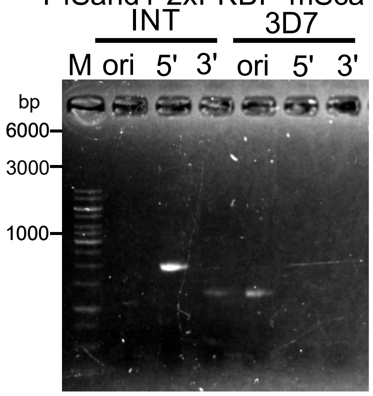

Figs 5D and S7 (experiment 1 in Fig 5D, experiment 1 - 3 in S7 Fig)

CoIP with PfRbsn5-2xFKBP-GFP-2xFKBP^{endo} + PfVPS45-mCh^{epi} parasites

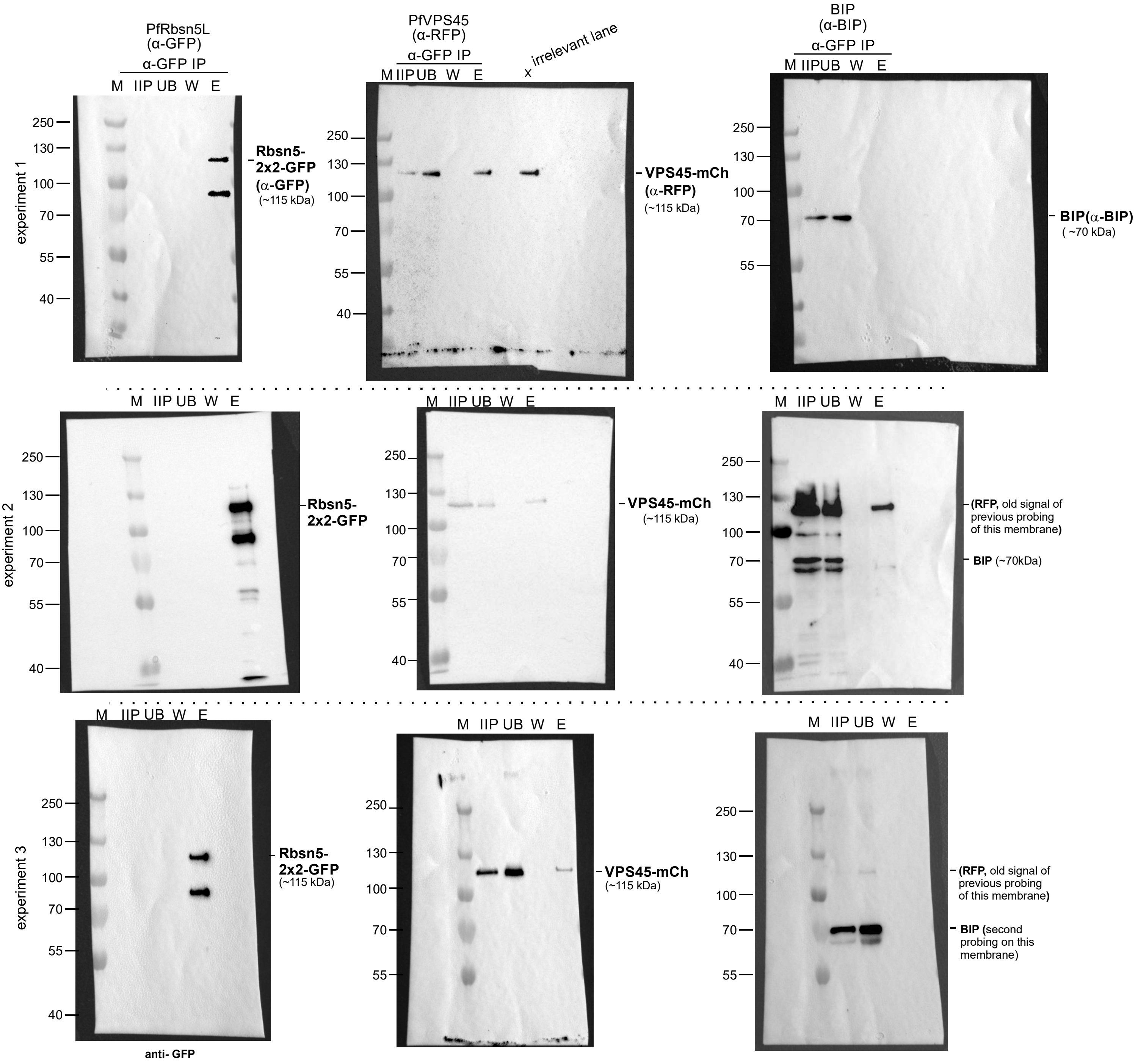

Figs 5E and S8 (experiment 2 in Fig. 5E, experiment 1 - 3 in S8 Fig)

CoIP with PfRbsn5-2xFKBP-GFP-2xFKBP[endo] + PfRab5b-mCh[epi] parasites

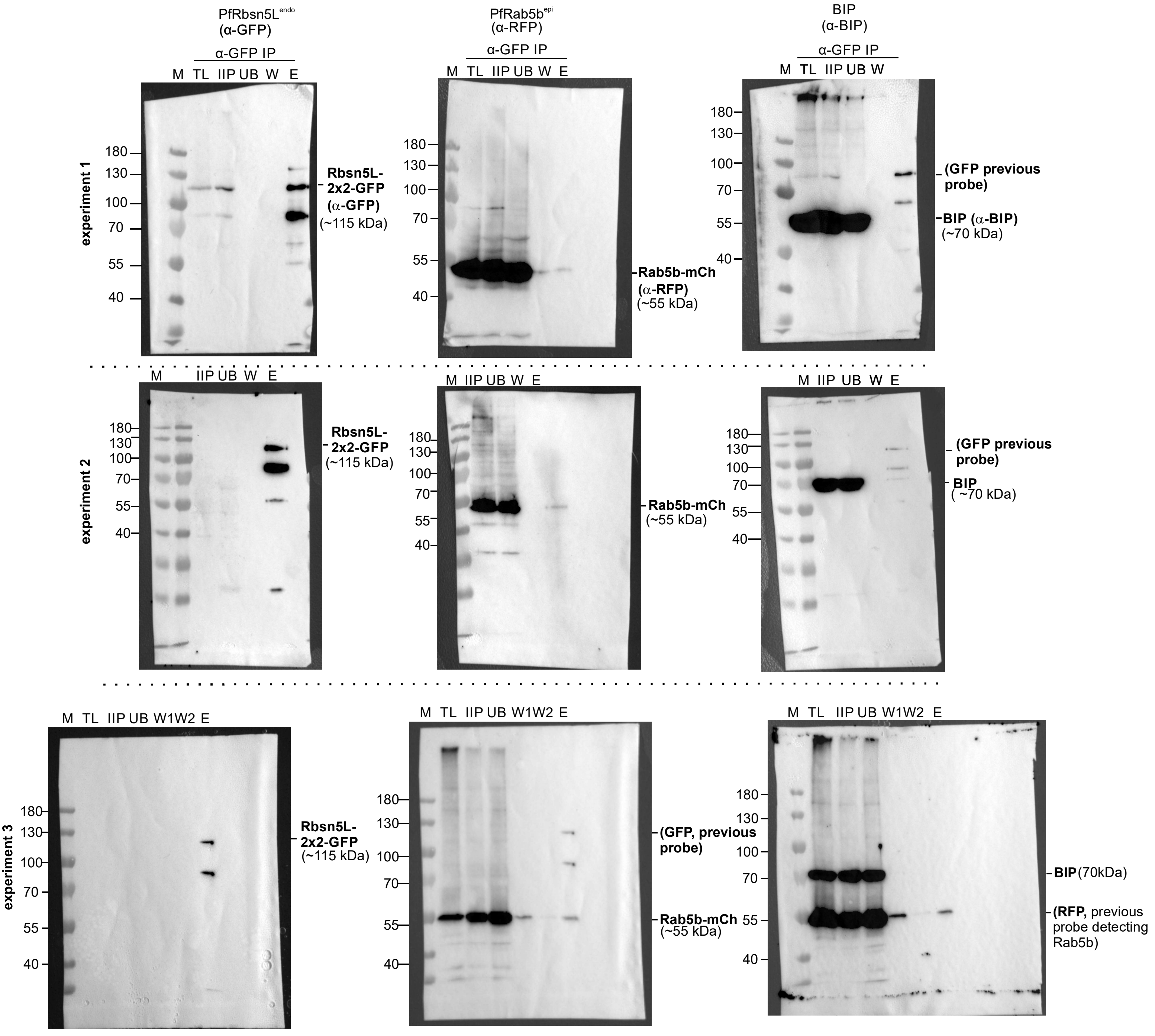

Supplement: S1 Raw Images — (PDF) [file pbio.3002639.s013.pdf]
