# Supplementary material for: Role of Rabenosyn-5 and Rab5b in host cell cytosol uptake reveals conservation of endosomal transport in malaria parasites
Source: PLoS Biol. 2024 May 31;22(5):e3002639. doi: 10.1371/journal.pbio.3002639 (PMC11168701; doi:10.1371/journal.pbio.3002639)
Supplement: Attachment — Submitted filename: Sabitzki_response to reviewers_2.pdf [file pbio.3002639.s014.pdf]

We thank the reviewers and editor for the time spent on reviewing this manuscript and their considerations to improve the manuscript. Please find our point by point response below in blue font.

Thank you for your patience while your manuscript "Identification of a Rabenosyn-5 like protein and Rab5b in host cell cytosol uptake reveals conservation of endosomal transport in malaria parasites" was peer-reviewed at PLOS Biology. It has now been evaluated by the PLOS Biology editors, an Academic Editor with relevant expertise, and by several independent reviewers.

In light of the reviews, which you will find at the end of this email,we would like to invite you to revise the work to thoroughly address the reviewers' reports.

As you will see below, the reviewers agree that the live fluorescence imaging is not of sufficient quality to justify the conclusions.

In particular,

we consider that it is important that you address the concerns regarding PI3P and rbsn5 co-localisation and the lack of an ER marker.

We now clarified issues regarding PI3P binding and co-localisation with PI3P positive areas of the cell, both experimentally and with *in silico* work. These findings indicate that interestingly the FYVE domain of PfRabenosyn5 does not bind PI3P (similar to a FYVE domain in a human protein which has previously been characterised (PMID: 23043110)) and shares key differences to other FYVE domains also found in the FYVE domain of PfRbsn5L). This explains why PfRbsn5L is not found uniformly at the food vacuole which is covered in PI3P, where the other FYVE domain protein EEA1 is located (PMID: 17289673) and which has the canonical PI3P binding motif. Nevertheless, we found consistent overlap of PfRbsn5L with PI3P positive regions in proximity of the FV (90% of cells) (extra data added to the manuscript), indicating that while it did not co-localizue with all PI3P positive areas, it was present at endosomal membranes, fitting with the observed function.

We now also did the co-localisation with an ER marker.

Reviewer #2 and #3 agree that Figures 2E and F require some correlation for P40 and rbsn5. We consider that given the high rbsn5 background staining this will be difficult with existing images.

We now provide extra images using confocal microscopy (to avoid out of focus light) that back up our conclusions on based on P40X and this clarifies the overlap of PfRbsn5L with PI3P: the results show that accumulations of PfRbsn5L overlap with PI3P directly in parts of the food vacuole or at P40 positive structures proximal to the food vacuole. However, consistent with the lack of PI3P binding of the PfRabenosyn5 FYVE domain (see previous comment, and below and new Fig. S1), the protein did not cover all PI3P positive regions, such as the entire FV. Nevertheless, this shows it is in regions where endosomal membranes are located. This is now presented as Fig. S4D.

We think that the authors indirect evidence of causation between rbsn5 KS and increased P40 staining vesicles is adequate to implicate rbsn5 in early endosomes with PI3P. We agree with reviewer #1 that the host cell cytosol in vesicles accumulating after PfRbsn5 KS in fig 2G is adequate evidence that the vesicles are derived from host cell endocytosis and we think that doing further experiments to prove the origin of the vesicles is not necessary.

We also agree that the vacuole bloating assay shows bloating in E64 treated parasites unless rbsn5 is KS, therefore we think that other comparisons are not strictly needed.

We thank the editor for clarifying these issues and responded to the corresponding reviewer comments accordingly.

We consider that it is not needed for you to remove results sections, eg KIC7, you can decide what parts of the story you are telling are important.

We thank the editor for this clarification. We actually believe this is a very important data point. We were not entirely clear why the reviewer thought it should be deleted but possibly because we inactivated two proteins at the same time and the system was not orthogonal (as indicated in the discussion of the original submission). We now added an experiment using chemical inhibition of endocytosis using cytochalasin D to give independent confirmation of this finding. These experiments confirmed our initial data, overall giving solid support for our conclusion that KIC7 and other previously identified endocytosis proteins lie upstream in the same pathway where the PfRbsn5L complex functions. Overall, we believe this not only strengthens the general model of haemoglobin endocytosis in the parasite but also provides further support for the role of PfRbsn5L in this process and for the first time provides experimental evidence for a link between the cytostome and downstream endosomal transport.

Please address the rest of the reviewers' issues.

Given the extent of revision needed, we cannot make a decision about publication until we have seen the revised manuscript and your response to the reviewers' comments. Your revised manuscript is likely to be sent for further evaluation by all or a subset of the reviewers.

REVIEWS:

Reviewer #1: Secretory and endocytic trafficking in malaria parasites.

Reviewer #2: Host cell-parasite interactions.

Reviewer #3: Cell signalling.

Reviewer #4: Malaria parasites biology.

Reviewer #1: In this manuscript, the authors convincingly demonstrate that a Plasmodium falciparum Rabenosyn-5 like protein and Rab5B are required for the delivery of hemoglobin containing vesicles to the food vacuole. They further show that the 2 proteins form a complex with PfVPS45, previously also shown by this group of investigators to be involved in the same process. Finally, they provide evidence using a very clever double conditional knock sideways assay that the PfKelch 13 compartment is upstream of the identified Rbsn5/VPS45/Rab5 complex.

This study is of broad interest for the readership of Plos Biology as it demonstrates that the pathway of host cell-cytosol uptake in the malaria parasite contains both parasite specific adaptations and features of a canonical endosomal system. The work is of high quality, novel and the paper is well written. All the data is provided in the manuscript and in the sup material.

We thank the reviewer for this very kind assessment.

Major comments:

1. -An important aspect that is not addressed much in the manuscript is the fact PfRbsn5 is likely a phosphoinositide binding protein due to the presence of a FYVE domain.

-Are the conserved PI3P binding attributes present in PfRbsn5? It would be important to show an alignment of the domain with well characterized FYVE domains, highlighting the conserved features.

We thank the reviewer for this suggestion, this was not sufficiently explored in our original submission. We now included an alignment of the FYVE domain of PfRbns5L with FYVE domains from other organisms and also for the tentative Rab binding motif (new Fig S1). In addition we used the AlphaFold2 predicted structure and compared it to experimental FYVE domain structures. Overall this analysis showed firstly, that the predicted structure is very similar to experimental structures of FYVE domains. Secondly, on the primary sequence level many of the signature residues are present. However, interestingly the sequence analysis also revealed that key residues for PI3P binding did not match, but resembled that of a FYVE domain known not to bind PI3P (but other PIPs) (see also next point). A larger alignment illustrating this is added here (Fig. S1 only contains very select sequences to make it more obvious but the large alignment here backs this up; Fig. S1 also contains a sequence logo on a large number of FYVE domains.

The first sequence in the alignment is PfEEA1, the second PfRbns5L.

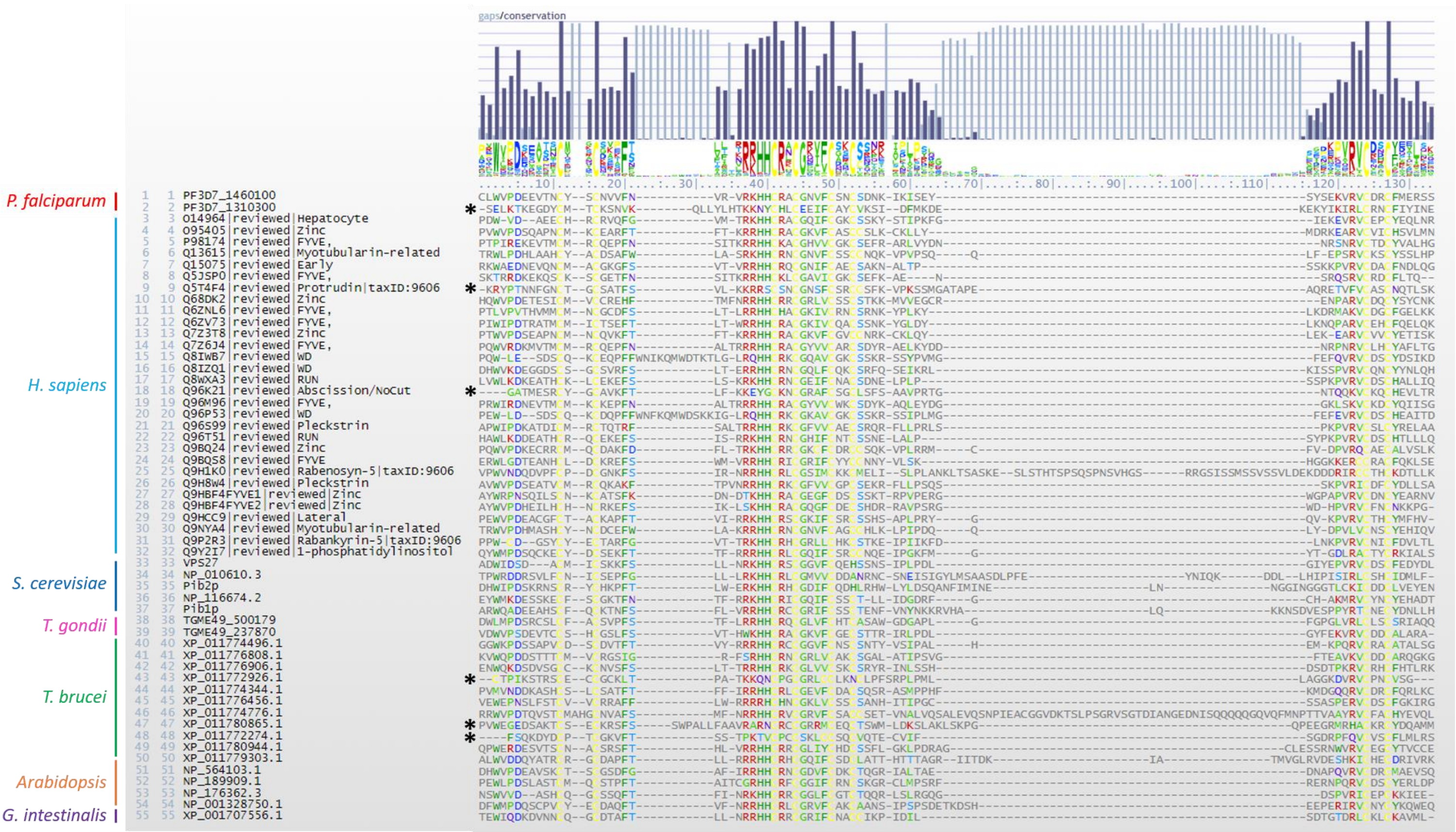

-It would be important for the authors to show whether the FYVE domain of PfRbsn5 actually binds PI3P. This could be done with a recombinantly expressed domain of WT and mutant domains and liposome binding assays. At the very least, the authors could episomally express in parasites a

WT and a mutant of the PfRbsn5 FYVE domain and see whether mutations of the putative PI3P binding residues abrogate the localization.

Given the known issues with PIP strips and the fact that liposome binding assays are not a trivial task and the issue that mutating such a small domain can well abrogate its folding (and hence would not show PI3P-binding specific loss of function), we opted to express the FYVE domain (using a 2xFYVE tandem) in the parasite. If it bound PI3P, this would be expected to lead to a location similar to the PI3P probe P40X that we used in the same cells. However, even though we use a tandem PfRbsn5L FYVE domains, the staining in the parasite was uniformly cytosolic and did not co-localise with P40X expressed in these parasites (new part Fig. S1D). In addition, we also expressed the tandem FYVE in mammalian cells. However, also there we did not find any association with cellular structures. As in the case of mammalian cells we can't exclude incorrect folding due to the heterologous origin of the sequence, and because the lack of binding in the parasite is much more conclusive, we did not include the mammalian cell data to the manuscript.

Overall, these findings fit with the sequence analysis (previous point) and indicate that the FYVE domain of PfRbns5L, at least by itself, does not bind PI3P. This might permit PfRbns5L to function upstream of the FV which is PI3P positive and would lead to full recruitment to its membrane. As PfEEA1 (FCP) is fully at the FV, it seems the two FYVE domain proteins of the parasite are located in partially different regions (see also new data supporting overlap of PfRbns5L with regions containing PI3P).

-If PfRbsn5 does bind PI3P, the authors should discuss where they think this occurs in the pathway. Does PfRbsn5 located on HCC vesicles bind PI3P on the food vacuole membrane or does it bind PI3P on the HCC vesicles? In the canonical endosomal pathway, early endosomes are labelled with PI3P which is then transformed in PI(3,5)P2 during maturation to late endosomes/multivesicular bodies. It is really intriguing that in Pf, PI(3,5)P2 has not been detected and that the food vacuole is massively labelled with PI3P despite being at a later/final step in the endosomal pathway. This is very different from model organisms. As such, I think that writing "reveals conservation of endosomal transport in malaria parasites" is too strong since only some elements are conserved. To be clear, I am not suggesting the authors try to answer this question in the current manuscript but since they are one of the (if not the) labs that have made the most critical contributions to the molecular understanding of the HCCU pathway in P. falciparum they certainly are in a position to speculate.

This is an excellent point and we fully agree with the reviewer’s take on this. We now toned down our message to "conservation of elements". The parasite most likely has a strongly adapted endosomal system where these more conserved elements (such as a Rabenosyn5L/VPS45/Rab5b complex) act. Furthermore, our results that the PfRabenosyn5 FYVE domain does not bind PI3P may provide a solution to how it can still function in an endosomal pathway where PI3P is present at the final destination, not at an intermediate on the way. If PfRbns5L bound PI3P, it likely would not be able to carry out its function in the transport step before the FV. This is now also treated in the discussion.

2. In Fig. 5A, whilst there clearly is some overlap between Rsbn5 and Rab5B, is it not 100%. In addition, in 5E, only a very small amount of Rab5B is pulled down by Rbsn. Furthermore, in 5H, Rab5B is not mislocalized in the Rnsb5 KS. This suggests that only a small fraction of total Rab5B complexes with Rbsn5. Can the authors comment on this? I'm not sure it is only a question of stability of the interaction as stated by the authors. This could be answered by crosslinking the

parasites before performing the pulldown. If stability is the cause then much more Rab5B should then be pulled down under these conditions.

Rab5b is also on other membranes (we now also added images co-localising it with an ER marker, see Fig. S5A), indicating that only some of the Rab5b population is in contact with VPS45-PfRbns5L. Rabs function through their interaction with effectors, for instance tethering factors which then lead to proximity of two different membranes which lead to fusion mediated by Sec1/Munc18 proteins and SNAREs. It is therefore likely that once the Rab5b-VPS45-PfRbns5L interaction is established the fusion reaction takes place and is not a constant complex. In contrast the VPS45- PfRbns5L interaction seems to be more permanent.

We were hesitant to do pull downs with crosslinked parasites as this would be complicated to interpret. It might better capture weak interactions, but it is unclear how this relates to the expected transient nature of the full fusion complex during function (it will stabilise and capture more of the interaction which then also will increase recovery). It likely would also increase yield for any interaction, independent of actual interaction strength. Given the ambiguity in interpreting a higher recovery after crosslinking we did not carry out this experiment. We however added to the main text the observation that less Rab5b was recovered compared to VPS45 in the IP.

Minor comments;

-In the title of Fig 3, I think it would be better to not use the HCCU abbreviation but instead use host-cell cytosol uptake.

Changed as suggested

Reviewer #2: In their manuscript, the authors have investigated the function of a rabenosyn 5 (RBsn5) homolog in Plasmodium falciparum (PfRbsn5) acting together with VPS45 and Rab5 in the endosomal vesicular transport of host hemoglobin to the food vacuole. Through gene/protein expression manipulation, they conclude that hemoglobin uptake involves these proteins, with some parasite specificities (Kelch) and conserved mechanisms for a process of endocytosed material into a cell -as it is known in other organisms that Rbsn5, VPS45 and Rab5b function in one complex. Overall, data are well-presented, sometimes overinterpreted but their innovation is somehow limited. Some controls are missing.

We thank the reviewer for their overall positive assessment.

Figure 1A: the sequence of rabenosyn 5 (RBsn5) homolog in P. falciparum is shown but several differences in domains are noticeable with human or yeast RBsn5/Vac1, with such a low sequence conservation, makes the designation of this protein as PfRbsn5 perplexing. The choice of another name reflecting 'RBsn5-like protein' seems more appropriate.

We agree with the reviewer and now call the protein Rabenosyn-like, or short PfRbsn5L (see also added sequence analysis in Fig. S1 and response to sequence motifs to reviewer 1).

Figure 1B: the food vacuole revealed by hemozoin content on DIC images does not contain PfRbsn5 but PfRbsn5 is in juxtaposed vesicles (purple arrow). The sentence: "a signal was present at the food vacuole in trophozoite stages (Figure 1B, dark blue arrow) with a more intense focus at the food vacuole in schizont stage parasites (Figure 1B, purple arrows)" is overstated.

What is the authors' interpretation about PfRbsn5 signal detected in the nucleus (light blue arrow) at young blood stage in Fig. 1B and 1C- like the mislocalizer (nmd3'1xNLS-FRB-mChepi)?

In regards to the nuclear signal: this is an intriguing point and we do not known what this signal means. It could be that Rbns5 has an additional, so far unknown function. The haemoglobin-filled vesicles clearly indicate that there is an endocytosis phenotype and given the speed of appearance and the evidence for a complex with VPS45 and Rab5b that do not show a nucleus location indicate that this phenotype is the cause impacting parasite growth (see also new data on death phenotype which indicates a catastrophic congestion with vesicles in new Fig. S4C and S5D), we do not think the nuclear pool is directly affecting the main topic of this paper. It is however interesting that the FYVE domain that has similar altered signature amino acids as PfRbns5L appear to have a tendency to go to the nucleus (PMID: 23043110). In addition there is at least one FYVE domain protein that is located at the microtubule organising centre and appears to function in a checkpoint between chromosome segregation and ESCRT function in abscission (PMID: 24814515). As any direct relation to this is rather speculative, we did not further discuss this in this work.

In regards to the food vacuole associated pool of Rbsn5 being overstated we apologise for the imprecise wording in regards to the purple arrows: we amended the text to more clearly allow for the fact that the purple arrows indicate accumulations in proximity of the FV. More evidence for the food vacuole proximal signal can now be found in new Fig. S4D (see response to point on Figure 2E below). There are two different types of signals, firstly one that appears to be directly at the FV membrane (circular structure around the hemozoin and what is also shown in Fig. 1B; frequently this is however much less uniformly around the FV than e.g. the PI3P probe, see Fig. S4D) as well as accumulations proximal to the FV that again frequently overlap with PI3P regions (Fig. S4D). The example in Fig. 1B on our computer screens seems clearly at the FV. We do not think this is an overstatement (we are unsure if the reviewer was also referring to the blue arrow, as this is part of the quote used). To illustrate this, we here added several line plots in various directions through the cell with the blue arrow of Fig. 1B to illustrate this. Please note that the directionality of the line plot is not always left to right. Some of the lines were also done through one of the nucleus proximal foci, to give a relative comparison. Although the maximum intensity at the FV is less than at the nucleus-proximal foci, it distributed over a more extended area. The total amount of PfRbsn5L at this site is therefore likely higher.

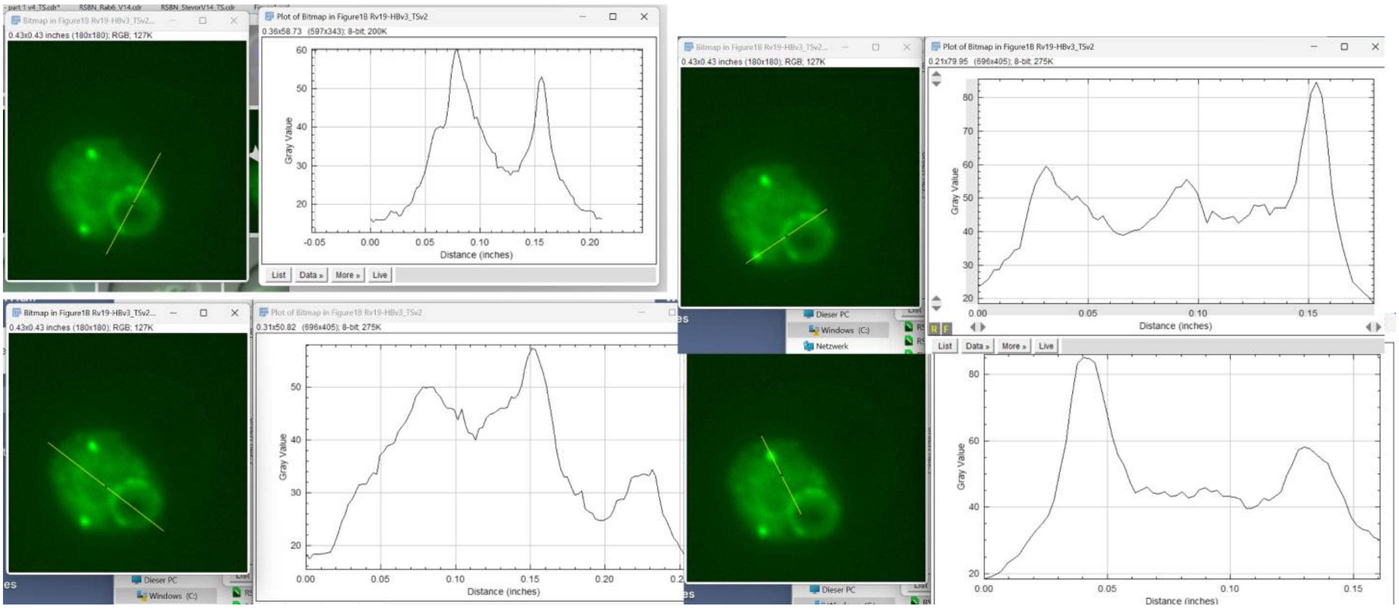

We also noticed that the GFP/DIC overlays in Fig. 1B were of poor quality and this has now been amended.

Figure 1C: the source of fluorescently tagged Graspepi /plasmid is not indicated. Same for mScarlet tagged P40PX in Figure 2E.

The P40X plasmid is in the plasmid file (crt'-p40-mSca_nmd3'-NLS-FRB-T2A-DHODH). The GRASP plasmid has previously been used and this is now mentioned in the materials and methods. There is now also mention of the additional plasmids used for the revision.

Fig. 2A mirrors data I Fig. 1F but a clarification for the origin of these vesicles would be informative with an immunostaining with a PPM and PVM markers to assess their formation through endocytosis. Same comment for Figure 3.

We see the point of the reviewer but given the fact that these vesicles contain haemoglobin (and dextran when it is loaded into the host cell) and no haemoglobin reaches the FV when the vesicles occur, the origin of the content of the vesicles is in our view not in doubt and hence also must derive from said membranes. Please also note that the bloated FV assay gives directionality, as only upon E64 addition haemoglobin accumulates in the FV, excluding that the phenotype observed arose from a fragmentation of the FV. Tracking the specific membranes is not trivial, as apart from some limited data on MSP1, there is so far no protein know that would mark the internalised membrane (integral membrane proteins might be excluded) and to our knowledge no such tracking has so far been achieved. We would like to note that the editor indicated this experiment is not strictly needed.

Fig. 2B misses a control for food vacuole size of untreated, WT parasites to compared with Rbsn5-deficient parasites exposed to rapalog and E64. Same comment for Figure 3.

This assay in this form does not compare FV size, it shows % of parasites with a bloating phenotype (filling up of the FV due to accumulation of haemoglobin) which does not occur anymore when PfRbns5L is inactivated. This is very obvious and scoring % of cells with or without bloated vacuole in control vs rapalog if hemolobin uptake is so profoundly inhibited is very straight forward.

Or is the reviewer referring to doing the assay with a control, i.e. 3D7 parasites? We did not do this because we have a much more stringent control: knock sideways with another essential protein that does not impact endocytosis and does not show a significant change in % of cells without bloated FV when rapalog and E64 is present. The same applies to Fig. 3. In addition, we had done 3D7 controls in the past and it shows no effect (see PMID: 38039338) and now did it again for a recent publication (PMID: 38039338).

Fig. 2E is important to assess PI3P at the PfRbsn5 localization. To this reviewer, the signal for PI3P and Rbsn5 colocalization (yellow arrow) is not too convincing. Instead of showing the overlaid images on the DIC, it will be better to show the merge of the green and red signals with quantification (PDM+ values).On some images the green signal seems smaller that the red one. What is the meaning of the blue arrows relative to Rbsn5 mislocalization (which is located in the nucleus)?

The primary purpose of this experiment was to show that the vesicles that occur after induction of PfRbsn5L inactivation contain PI3P and that is when we realised that the FV proximal pool of PfRbsn5L is an area where PI3P is located. We see the importance of illuminating this better and that these images are not sufficient to show this point. To amend this, we increased the overall

intensity of some of the images in Figure 2E to make this more obvious. Secondly, we provide the merge requested by the reviewer. Thirdly, we did a more comprehensive analysis of PfRbsn5L with PI3P, particularly also in light of the finding that the PfRbsn5L FYVE domain does not seem to bind PI3P. For these imaging experiments we used confocal microscopy as the reduced interference of out of focus light permits a clearer comparison as the PfRbsn5L signal background is then less in a given focal plane. These experiments showed that PfRbsn5L overlapped with regions containing PI3P signal in almost all cells (48 of 53 analysed cells; this is now mentioned in the main text and images are shown in Fig. S4D). It also showed that the signal only overlapped with part of the PI3P signal.

The blue arrows indicate P40 signal accumulating in the area where the vesicles due to PfRbsn5L inactivation are (see figure legend: “Blue arrows: accumulations of PI3P near the food vacuole.”).

Figure 4D: the author must clarify the origin of the membrane pointed with the blue arrow to assess this is derived from the PVM. Or at least membrane pointed with the yellow arrow being the PPM as the parasite seems pretty messed up with high vacuolization upon all the treatments. For example,the membrane ofthe food vacuole is not apparent.

Please see response to comment on Fig. 2A on the question of the origin of the indicated membranes.

In regards to the cells being messed up. The main purpose of this experiment was to show that there is no connection to the outside left, which would result in empty vesicles which was not the case. Analysis of the integrity of the cellular structures is therefore better observed in the non-saponin treated cell, see Figure 2.

Figure 5B, F, I for the control conditions (no rapolog) are not convincing to assess Rab5 and Rbsn5 colocalization at this resolution, in contrast to VSP45 localization with either Rab5 or Rbsn5 more compelling.

Fig. 5B: the overlap of Rab5b with Rbsn5L appears to be in small regions of the overall Rab5b signal (that is also in other membrane regions) and is either in foci proximal to the FV or at a “line” at the FV. In that respect we believe the cell shown in Fig. 5B is quite representative. We moved the arrow out of the way in the zoomed images of Fig. 5B and uniformly increased the intensity of these images somewhat to make this more obvious. We hope this is now better visible (see image pated below). In addition, we carried out confocal microscopy and now show 3 examples in the supplement (Fig. S6D-F) to illustrate the overlap phenotype (again the overlap is proximal to the FV).

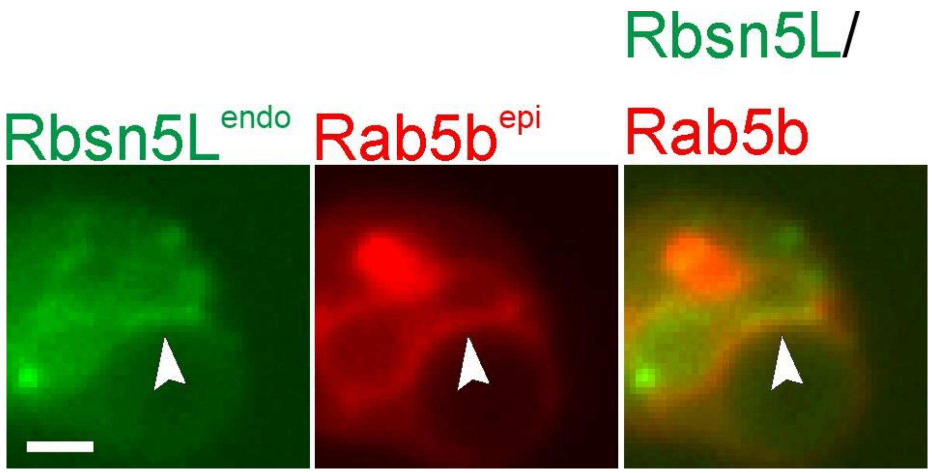

Our assessment is also supported when a line plot is done with the shown images. The line was drawn in ImageJ in synchronised windows starting from the top right (i.e. the first peak in green is the focus in green first encountered and where no Rab5b is). The major peak is the area of overlap indicated by the arrow in the figure.

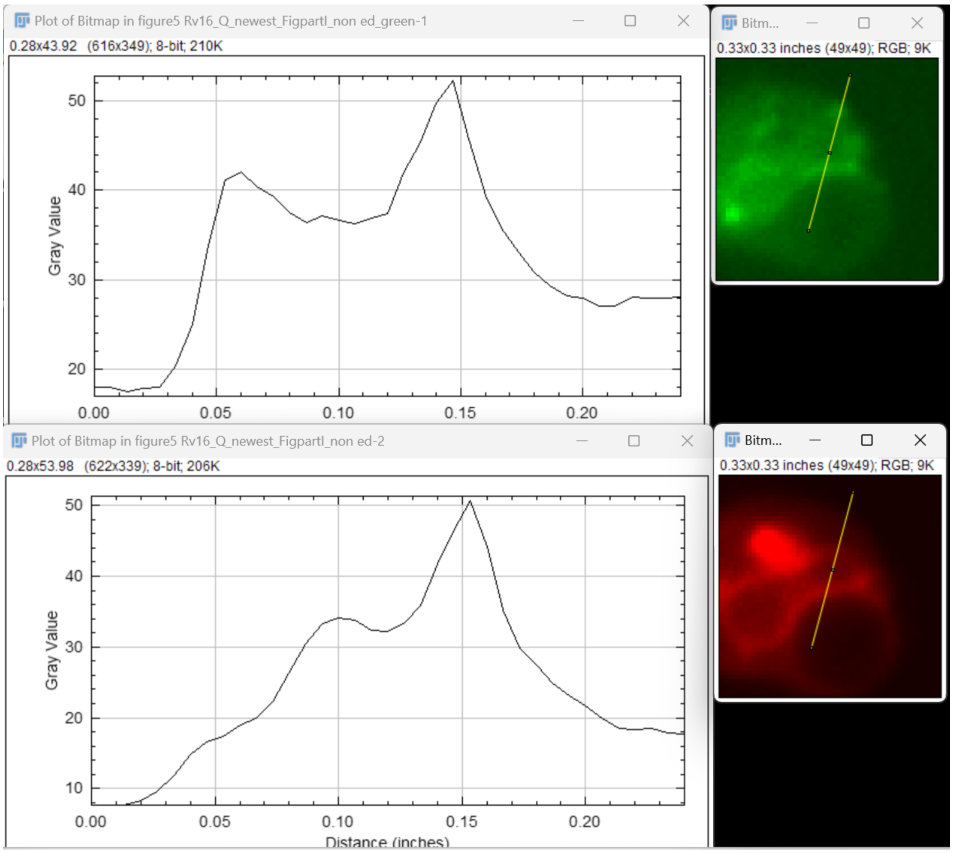

Figure 5F and I controls. The GFP channels of these images had been of low intensity. We uniformly increased the intensity of these channels. Note that in Fig. 5F, the food vacuole located PfRbns5L focus is just barely detectable above background, likely because the microscopy focus of the image was more in the plane of the nucleus proximal PfRbns5L foci. We hope these images now more clearly show the distribution of the protein

Figure 6 about the cytostomal origin of the rbsn5 positive vesicles should be placed after Figure 4.

We tried to do this but it turned out to be slightly confusing to go back to the vesicle fusion complex after moving to the more overarching data in Fig. 6 (Fig. 6 places the process in the entire endocytosis pathway and provides experimental evidence for to connect it to the cytostomal function). Hence, we ended up placing this figure back where it had been and hope this is acceptable.

The Discussion section is more or less a summary of the Results and could be combined.

We have to admit that we are not that much in favour of combining the discussion with the results. We do discuss where the fusion complex could function (direct fusion event to FV or laterally such as thought to occur in yeast with VPS45) and also the stage specific differences in function which would be complicated to do if combined with the results. We therefore opted to keep the organisation as it is.

Reviewer #3: This work described in this manuscript by Sabitzki et al. attempts at understanding the role of small GTPase PfRab5b and its putative homologue of Rabenosyn 5 (PfRbsn5) in the malaria parasite. A reverse and forward genetics approach is taken for these studies, which involves the conditional inactivation of these two and other related proteins and these mutants are mainly used to evaluate the process of host erythrocytecytosol-which is mainly composed of haemoglobin-uptake(HCCU). Previously, PI3K/VPS34and VPS45 have been demonstrated to play a role in this process in malaria parasite. Given that Rab5 is known to be an effector of endocyticpathways regulated by VPS34 and VPS45 in yeast and mammals, the finding that PfRbsn5 and PfRab5b are also involved in HCCU were not surprising. I feel in general the study is nicely organized and experiments have been planned properly and executed effectively.However, at times authors have over-interpreted the data and need more experimental evidence to support some of the conclusions.
We thank the reviewer for their overall positive assessment.

Specific comments/queries:

1. Fig. 1A. The sequence comparison between PfRabsn5 with homologues indicated a lack of several key elements like C2H2 motif. It is important to provide the sequence comparison between the FYVE and Rab binding domains of various homologues.

This is an important point also raised by the other reviewers. These data are no provided as a new Fig. S1 (see response to reviewer 1 and 2).

2. Given that PI3P is a key player in endocyticprocesses like HCCU and PfRabsn5 has a FYVE domain, it is extremely important to demonstrate that PfRabsn5 interacts with PI3P via its FYVE domain and its cellular localization is dependent on this interaction. These experiments are essential for this study.

See our response above to reviewer 1 for a more details. Expression of a tandem of the PfRabsn5L FYVE domain did not result in any apparent recruitment to PI3P positive membranes (Fig. S1D). Furthermore, assessment of the PfRabsn5L FYVE domain indicated important differences to the common consensus that are also present in the FYVE domain of protrudin that does not bind PI3P. In addition, we substantiated the co-localisation of PfRabsn5L with some but not all regions containing PI3P (Fig. S4). Overall this indicates that PfEEA1 (FCP) with a canonical FYVE domain is fully at the FV (PMID: 17289673) while PfRabsn5L only partially overlaps with PI3P positive areas which might indicate that it functions at a fusion event before or from a donor with the FV which might not be possible if it bound strongly to PI3P and be sequestered to the FV.

3. Fig. 1C. Co-localization between Rbsn5 and Golgi marker GRASP is suggested. While some overlapping localization is observed, better images are needed to make this point. It is important to perform co-localization with an ER marker. Typically, Golgi is not known to contain much PI3P andis mainly rich in PI4P.

It is important to note that PfRbsn5L is not regularly fully overlapping with the Golgi, we now clarified this in the manuscript main text and provide a quantification in Fig. 1C. In addition, we provide line plots in Fig. S2B to illustrate the foci of PfRbsn5L that overlap or not with GRASP. This also illustrates that some of the foci are less prominent. We believe these foci are clearly visible in the PDF version of the supplemental figures before upload but hope that the line plots further illustrate this. Hence, while PfRbsn5L foci can be seen in the proximity or overlapping with the Golgi, this is not the most prominent location. We also provide co-localisation with the ER (Fig.

S2C, now mentioned in the main text) and found that apart from the nucleus proximal focus, there was no overlap with the ER, particularly of the food vacuole proximal pool of PfRbsn5L. As the ER covers a large area of the parasite, we provided 4 image series to illustrate this point.

4. Figure 1E. The inactivation of PfRbsn5 impairs asexual development of the parasite. It is important to find out which stage and the process of parasite development is regulated. A more detailed analysis of the mutant is needed to address this issue.

We now carried out detailed analyses on the growth phenotype in synchronous parasites and this is included as new Fig. S3 and Fig. S4C. This showed that PfRbsn5L inactivation in rings prevents most parasites from turning into trophozoites. If inactivated later (or a smaller proportion of the rings continuing in the cycle from the ring stage-induction), leads to a severe phenotype in the trophozoite to schizont stage. It is not trivial to pinpoint what exactly causes death. The arrest of the rings might be similar to endocytosis inhibition of Kelch13 compartment proteins which prevents the parasites from entering the trophozoite stage (PMID: 28288121, PMID: 31896710). In the trophozoites, the parasites fill up with the vesicles containing host cell cytosol which likely causes all sorts of problems. Some parasites lyse, some appear to consist almost entirely of the vesicles without recognisable typical morphological features. To illustrate this, we provide images showing the state of the parasites from the synchronous growth assays in DIC (Fig. S4C).

5. Fig. 2B/C. It is important to indicate the stage and/or time after rapamycin additionat which parasites used for the experiments reportedin this and other figures.

This information is already present (8h rapa is indicated in the figure). In addition, in response to this comment, we now added a referral to the materials and methods where the details are mentioned: "Bloated food vacuole assays were performed as described [13]. The respective mixed parasite culture was synchronized twice with 5% sorbitol at 10 h intervals to obtain a ring stage parasite culture with a 10-18 hours post invasion stage window. The parasites were cultured for 8 hours (resulting in a stage window of 18- 26 hours post invasion) and divided into two 1-ml dishes to which E64 protease inhibitor (Sigma Aldrich) was added to a final concentration of 33 µM. One dish was additionally treated with rapalog (250 nM), while the other served as control. The parasites were cultured for 8 hours, stained with 4.5 µg/ ml DHE for 20 min at room temperature, washed once in RPMI, and imaged…"

6. Figure 3B. PfRab5b is reported to be presentat the food vacuole and also at the ER. While food vacuole localization may be acceptable due to the presence of hemozoin pigment, co-staining with an ER marker is needed to conclusively to prove ER localization.

The ER of the parasite consists of the nuclear envelope with "horns" which is quite a typical pattern. We now generated a parasite line expressing an ER marker in the Rab5b parasites to confirm that Rab5b is also at the ER. This has been added as Figure S5A.

7. Figure 3D. PfRab5b depletion arrests parasite development but again the exact stage of development is not reported, which needs to be done.

This has now been addressed as indicated for point 4 and the data is provided as Figure S5C. No effect on rings was observed after Rab5b inactivation and the ring induction was therefore used to also assess the effect on later stage development. The results in later stages were similar to PfRbsn5L inactivation but agreeing with the lower number of vesicles and lower severity of the phenotype, was less pronounced. We also observed a similar phenotype indicative of vesicle congestion in parasites at the time points the growth phenotype was apparent (Figure S5D).

8. The co-localization between PfRab5b and PI3P- reporter p40 is very evident but it is not the case with Rbsn5.

We now provide confocal images to address this point as indicated to reviewer 2 (comment on Figure 2E) (see Fig. S4D).

9. In my opinion, KIC7 related experiments do not add much to the story and they can be left out.

It is unclear why the reviewer thinks that, in our opinion these experiments are very important (see also comments on this by reviewer 1). Maybe the reviewer has some doubts because there is some limit to the system as we used the same inactivation system? To amend this, we now also included experiments using cytochalasin D, which inhibits endocytosis in trophozoites and leads to accumulating host cell cytosol filled vesicles in the parasite (PMID: 25724884; PMID: 18477610). Taking advantage of this chemical inhibition, we repeated the experiment inactivating KIC7 which showed that if KIC7 was inactivated, no Cytochalasin D-induced vesicles occurred whereas if KIC7 was still functional, they did. Again, this shows that the Kelch13 compartment function lies upstream of this event when a different type of inhibition was used than PfRbsn5L inactivation. This has been added as Fig. 6F, G. In addition, we added a simply scheme, to show the experimental setup for these experiments to Figure 6 in an attempt to make this more accessible.

10. General Comment: the images provided in this study are not of a very high quality, which has to be the case as manuscript deals with issue of vesicular trafficking . Since live imaging was reliant on GFP/mcherry fluorescence, it is indeed possible that low levels of expressions of the protein may contribute to suboptimal imaging in some cases. It may be worth attempting IFAs using antibodies against GFP/mcherry in addition to live imaging.

PfRbsn5L indeed poses some challenges for imaging as it is at multiple sites in the cell and is of rather low expression. We attempted to better illustrate the distribution of the proteins in the current version of the manuscript and hope we were able to substantiate the key imaging findings. Vesicle trafficking proteins imaged in other systems (e.g. mammalian cells) often also have wide distributions in the cell, sometimes with soluble backgrounds. Often such proteins shows merely an enrichment on a membrane, in taking with the transient nature of association of individual proteins at the membrane that is inherent to their function. We believe this is rather similar with some of the imaging in this manuscript.

IFAs: after many years of experience with IFAs and live cell imaging we would very much prefer to stay with the live images. IFAs are complicated for many reasons: accessibility of antibody and morphology are inversely correlated and may not be uniform between cells and even within one cell. Soluble pools of proteins (or less well-fixed pools) often leak out of the cell, resulting in non-physiological representation (we have observed this frequently for instance with proteins soluble in the host cell or in the parasite cytoplasm that can appear in a dot pattern by IFA when live this is a uniform pool in live cells). While such experiments might show stronger overlaps at fixed cellular sites, we always worry this may not be the true physiological representation and if possible, opt for live cell imaging which should show overlaps, if there are any. We believe the live images in this work do show these overlaps and hope this is now better visible.

Reviewer #4: As usual, manuscripts from this laboratory are always of a high scientific and intellectual quality. I must admit that I am still suffering from a bout of Covid-19 and may have overlooked some small mistakes, however I have a generally very positive opinion of this manuscript and it's significance. I must apologise for the briefness of this report, I really am not in the best of health.

We thank the reviewer for their very kind assessment and hope they fully recovered.
