# Supplementary material for: Role of Rabenosyn-5 and Rab5b in host cell cytosol uptake reveals conservation of endosomal transport in malaria parasites
Source: PLoS Biol. 2024 May 31;22(5):e3002639. doi: 10.1371/journal.pbio.3002639 (PMC11168701; doi:10.1371/journal.pbio.3002639)
Supplement: Attachment — Submitted filename: Sabitzki et al Response_R2.pdf [file pbio.3002639.s015.pdf]

Response to editorial and reviewer points (in blue font):

Editorial points:

a) Please change your title to the following: "Role of Rabenosyn-5 and Rab5b in host cell cytosol uptake reveals conservation of endosomal transport in malaria parasites"
The title was changed as indicated.

b) Please attend to the remaining requests from reviewer #1. You'll see that reviewer #2 is satisfied.
All comments of reviewer 1 were addressed.

c) I discussed the requests from reviewer #3 with the Academic Editor, who said "The authors have been suitably cautious in describing their findings but they should address the reservations of R3 by inclusion of text in the introduction clarifying that the binding specificity of PfRbsn5L remains unknown. Their transformation of parasites with recombinant FYVE domains that do not colocalise with PI3P doesn't prove PfRbsn5L doesn't bind PI3P but I accept that this proof is not trivial and could be itself an independent publication." Thus, while we understand that these experiments would greatly strengthen the study, addressing these concerns textually will be acceptable for publication at this stage.
We thank the editor for giving this guidance on how to address the remaining concern of reviewer 3. We changed the introduction accordingly and inserted that we do not know the binding specificity of PfRbsn5L as follows (added text in bold): "Our data provide evidence that the *P. falciparum* Rab5-Rbsn5-VPS45 fusion complex - and thus elements of this part of the endosomal pathway - is evolutionarily conserved **although the binding specificity of the PfRbsn5L FYVE domain remains unknown.** ". In addition, we added a section in the discussion following reasoning suggested by reviewer 1 that further treats this point.

d) Many thanks for providing the underlying data in S1 Data. Please cite the location of the data clearly in all relevant main and supplementary Figure legends, e.g. "The data underlying this Figure can be found in S1 Data."
We now added a reference to S1 Data in all relevant figure legends.

e) Please make any custom code available, either as a supplementary file or as part of your data deposition.
No custom code was used.

As you address these items, please take this last chance to review your reference list to ensure that it is complete and correct. If you have cited papers that have been retracted, please include the rationale for doing so in the manuscript text, or remove these references and replace them with relevant current references. Any changes to the reference list should be mentioned in the cover letter that accompanies your revised manuscript.
The reference list was checked once more. We are not aware of any retractions of the cited papers. We added one more citation in response to reviewer 1 (PMID: 29154995) and in the added part cited another two publications that already were in the reference list.

REVIEWERS' COMMENTS:

Reviewer #1:

In this revised version, the authors have done an excellent job with providing additional data to support their conclusions. They have also reworded some sentences to better highlight that Pf has conserved some aspects of a canonical endosomal system but that there are still important differences. I have only very minor comments and recommend publication of this high quality piece of work.
We thank the reviewer for this kind assessment.

Comments:

1- -The absence of PI3P binding of the PfRbsn5L FYVE domain is a really interesting result and it is possible that, as suggested by the authors, it could bind other PIP species like the FYVE domain of Protrudin. I think it would be important to specify that the latter binds to PI(4,5)P2, PI(3,4)P2 and PI(3,4,5)P3 and comment on what potential PIP species the PfRbsn5L FYVE domain could bind to. Ebrahimzadeh et al (PMID: 29154995) had previously reported that overexpressing sensors specific to either PI(3,4)P2 or PI(3,4,5)P3 in P. falciparum blood stages resulted in a broad cytosolic signal, like what was seen here with the PfRbsn5L FYVE domain. This potentially suggest that it could bind either one of these PIPs. Since a PI(4,5)P2 sensor was shown to strongly label the parasite plasma membrane and potentially the cytostome (PMID: 29154995), it is unlikely that the PfRbsn5L FYVE domain would bind this particular species.
This is a good argument. We now added a part to the discussion stating that the PfRbsn5L FYVE domain likely does not bind the PIPs that already were detected by sensors in PMID 29154995. We also added that Tawk et al (already cited elsewhere in the manuscript) detected PI(3,4)P2 and PI(3,4,5)P3 in infected RBCs but that these PIP species were not seen by the specific sensors in PMID 29154995. Hence, these particular PIP species might also not have been easily detected by the PfRbsn5L FYVE domain (assuming it had such a specificity), as the experiment shown in the manuscript with the double PfRbsn5L FYVE domain in effect is similar to a sensor overexpression experiment.

2-I have found a few instances in the text and figs where Rbsn5 was not changed to Rbsn5L.
We thank the reviewer for pointing this out, we now went through the manuscript again using the word search function to ensure all instances of Rbsn5 instead of Rbsn5L were caught and corrected. We also corrected a few instances where the abbreviation was Rbns5L instead of Rbsn5L.

3-In the finale sentence of the introduction, authors write:

"Overall, our data suggest that HCCU consists of a parasite-specific initial part at the PPM that delivers endocytosed material into a more canonical endosomal system."

I think it is important to specify that only some aspects are like the canonical endosomal system, several others are not, as discussed in the manuscript. It might seem like a trivial suggestion but I think it is critical for the readers to understand that not only the initial steps of HCC endocytosis contain parasite specific biology.
We changed this sentence to say that endosomal transport has “more canonical aspects”.

4- In the Discussion, the authors write:

"PfRab5bL has a role akin to that of Rab5 isoforms in other organisms"

I presume that they are talking about PfRab5b therefore the L should be deleted.
We thank the reviewer for pointing this out, this was amended.

5- In the next sentence, the authors write:

"However, there are also important differences. We did not find any evidence for PI3P binding of its FYVE domain"

I think "its" should be replaced by "the PfRbsn5L" for clarity.
We thank the reviewer for pointing this out, this was changed as suggested.

Reviewer #2:

This reviewer is globally satisfied by the authors improvements of this manuscript. The new confocal microscopy images provided have increased the plausibility of the findings.
We thank the reviewer for this assessment and going again through our manuscript.

Reviewer #3:

In this revised version of the manuscript by Sabitzki et al. , authors have tried to address issues raised by me and other reviewers. However, one of the major issues remains unresolved, which has also been raised by other reviewers:

I feel it is extremely important to demonstrate that PfRabsn5 interacts with PI3P via its FYVE domain and its cellular localization is dependent on this interaction. Similar queries have been raised by other reviewers as well. Authors have stated reasons like "issues" related PIP-strips and non-"trivial" nature of liposome assays for not performing these important assays. If done with proper positive and negative controls experiments with PIP-strips can be very informative and continued to be used. The authors have relied on in silico analysis and proposed that PfRabsn5-FYVE domain may resemble me related to non-PI3P binding FYVE domains. It is important to know if PfRabs5 binding domain interacts with any other PIPs especially when comparisons are drawn to protruding, which interacts with PI3P with less affinity in comparison to PI(3,4)P2 and PI(3,4,5)P3 (Gil et al. JBC2012). They have relied on the overexpression of 2XFYVE domain construct to suggest that it is not targeted to PI3P rich locations. It is possible that PfRabsn5-FYVE binds with less affinity with PI3P or does not interact with it at all; either way it needs biochemical demonstration.

We agree with the reviewer that the binding specificity of the PfRbsn5 FYVE domain is an interesting point and that we can't exclude with certainty it still binds PI3P. However, given the finding that (i) full length PfRbsn5L overlaps with only some of the PI3P positive areas, (ii) the overexpression of a doublet of its FYVE domain is not recruited to the PI3P positive areas, and (iii) the domain contains residues at odds with PI3P binding if structural

considerations from other FYVE domains are considered, there is reasonable doubt that it binds PI3P. Clearly, we can't exclude (weak) PI3P binding. We now further discuss this point in response to reviewer 1 and, based on the editorial suggestion, added a statement to the introduction that the binding specificity of the PfRbsn5L FYVE domain remains unknown.
